# Cardiac biomarkers in athletes and active individuals: a clinical review of exercise-induced elevations and diagnostic interpretation

**DOI:** 10.3389/fphys.2026.1868998

**Published:** 2026-07-13

**Authors:** Chaoming Wu, Yanshuang Zhao, Yanxia Zhao

**Affiliations:** 1 Chongqing Luneng Bashu Secondary School, Group of Physical Education, Chongqing, China; 2 People’s Liberation Army 96941, Beijing, China; 3 Chongqing University, College of Physical Education, Chongqing, China

**Keywords:** cardiac biomarkers, exercise-induced myocardial injury, galectin-3, high-sensitivity troponin, natriuretic peptides, non-coding RNAs, precision medicine

## Abstract

Exercise-induced increases in cardiac biomarkers, especially natriuretic peptides (B-type Natriuretic Peptide (BNP)/N-terminal (NT)-proBNP) and high-sensitivity troponin (hs-cTn), make it more difficult to distinguish between myocardial disease and physiological adaptation in athletes. The pathophysiology, release kinetics, and diagnostic performance of established biomarkers (hs-cTnI, hs-cTnT, BNP, NT-proBNP) and emerging candidates (galectin-3, ST2, GDF-15, heart-type fatty acid-binding protein, cardiac myosin-binding protein-C) are examined in this review. The main limitations include the lack of athlete-specific reference ranges (74–96% of marathon runners exceed standard hs-cTn thresholds), small sample sizes (most n<100), short follow-up, and varied exercise protocols. The assessment of acute cardiac stress, the distinction between an athlete’s heart and cardiomyopathy, and the choice to return to play after myocarditis continue to be the principal clinical uses. Without thorough confirmation, claims like artificial intelligence-powered multi-omics, tailored molecular exercise prescription, and real-time monitoring remain theoretical. Sport-specific 99th percentiles, large prospective cohorts with 5–10-year follow-up, standardized methodologies, and verified point-of-care testing are all necessary for future priority. Until then, prolonged increases (>72 hours) or symptomatic presentations warrant further research, whereas exercise-induced biomarker elevations in asymptomatic athletes should be considered mostly benign and temporary.

## Graphical Abstract

**Figure:**
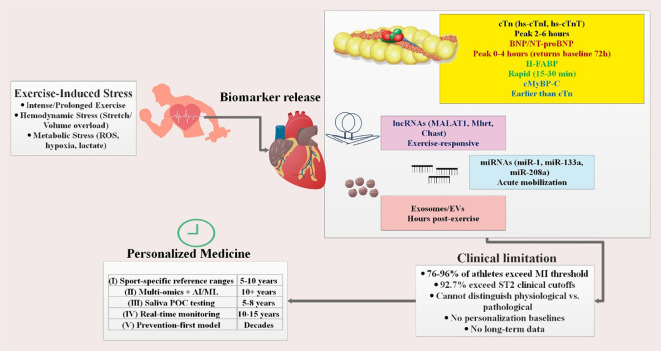
(1) Release kinetics of existing biomarkers (cTn, BNP, H-FABP, cMyBP-C) and novel biomarkers (miRNAs, lncRNAs, EXOs/EVs) during HES with hemodynamic/metabolic stress. (2) Clinical limitations: 76–96% of athletes surpass MI thresholds; no individualized baselines; insufficient long-term outcome data; inability to differentiate between physiological and pathological elevations. (3) Future goals with reasonable timeframes (5–10 years to decades) are all still theoretical without thorough verification (Ranjbar et al., 2017; Donnellan and Phelan, 2018; López-López and Pareja-Galeano, 2018; Peretti et al., 2018; Cirer-Sastre et al., 2019; Mahanty and Xi, 2020; Aengevaeren et al., 2021; Su and Chen, 2025; Zheng et al., 2025; Greco et al., 2026; Liu et al., 2026; Shen et al., 2026).

## 1 Introduction

Cardiovascular diseases (CVDs) continue to be a major cause of death worldwide, with notable regional differences. Asia’s age-standardized CVD mortality rate (542.3 per 100,000) is 1.8 times greater than the global average, and coronary heart disease (CHD) accounts for 337.4 deaths per 100,000. Global CVD fatalities are expected to surpass 23 million by 2030, and the high incidence of CHD in China puts a heavy financial and psychological strain on patients and their families (Bo et al., 2026; Li et al., 2026). The incidence of CVD is still rising despite improvements in diagnosis and treatment, highlighting the need for new approaches. Frequent exercise is essential for preventing CVD, although the exact molecular mechanisms underlying its cardioprotective benefits remain unclear (Bo et al., 2023; Abdelaal et al., 2025; Bo et al., 2026; Sun et al., 2026). Physiological cardiac adaptations induced by prolonged exercise, such as an enlarged left ventricle and increased muscle mass with normal function, usually reverse when training is stopped. However, extreme endurance training has been linked to myocardial fibrosis, especially in the right ventricle (Sanchis-Gomar et al., 2022). Poor cardiorespiratory fitness (CRF) is a significant risk factor for CVD, while high CRF denotes low morbidity and mortality. CRF is a potent predictive tool, as one metabolic equivalent increase lowers the risk of CVD by 15% (Levine, 2014; Sanchis-Gomar et al., 2022). Exercise training (ET) also enhances autonomic function, increasing heart rate variability, causing resting bradycardia, and guarding against potentially fatal arrhythmias (Kodama et al., 2009; Hillebrand et al., 2013; Sessa et al., 2018; Sanchis-Gomar et al., 2022). The sports cardiologist plays a crucial role in differentiating physiological adaptations from pathological changes on electrocardiogram (ECG) and imaging, integrating clinical history with diagnostic testing, and employing a patient-centered, shared decision-making approach for risk stratification and return-to-play decisions (Martinez et al., 2021).

This review focuses on a single clinical inquiry: How can doctors differentiate benign physiological adaptations from pathological CVDs in athletes and active adults who exhibit exercise-induced increases in cardiac biomarkers?

Because they may resemble acute myocardial infarction (AMI) or heart failure (HF) in athletic populations, exercise-induced increases in cardiac biomarkers, especially high-sensitivity troponin (hs-cTn) and natriuretic peptides (NPs) (B-type NP (BNP)/N-terminal (NT)-proBNP), pose a substantial diagnostic challenge (Scharhag et al., 2008; Tomassetti et al., 2026). High false-positive rates and possible misinterpretation result from the current diagnostic thresholds, which were developed for sedentary populations and are not optimal for athletes. For instance, after endurance activity, 74% to 96% of healthy marathon runners exceed the traditional 99th-percentile upper reference limit (URL) for hs-cTn, and 92.7% exceed clinical cutoffs for suppression of tumorigenicity 2 (ST2). Differentiating between benign, physiological biomarker increases and actual pathological harm in athletes exhibiting dyspnea, chest discomfort, or other cardiac symptoms presents a crucial therapeutic challenge. For diagnostic interpretation, it is crucial to comprehend the different release kinetics: exercise-induced cTn usually peaks 2–6 hours after exercise and returns to baseline within 24 hours, whereas NT-proBNP peaks later and stays elevated up to 72 hours, in stark contrast to AMI, where cTn remains elevated for days (Bansal et al., 2015; Kong et al., 2017; Legaz-Arrese et al., 2017; Cirer-Sastre et al., 2021; Papamichail et al., 2023).

Traditional cardiac biomarkers (troponins and NPs) proficiently detect myocardial necrosis and distension. Nevertheless, they are unable to distinguish between pathological damage and physiological adaptation, nor can they provide a mechanistic understanding of fibrosis, inflammation, or extracellular matrix remodeling, all of which are critical processes in exercise-induced cardiac modifications. Emerging biomarkers, such as soluble ST2 (sST2) and galectin-3 (Gal-3), provide further insights. Both exhibit significant increases post-exercise, indicating measurable alterations in fibrotic and remodeling pathways overlooked by traditional markers (Goff, 2023; Lyu et al., 2024). sST2 signifies myocardial strain, inflammation, and matrix remodeling, while Gal-3 denotes fibroblast activation and collagen deposition (cardiac fibrosis). These novel biomarkers provide mechanistic specificity, enable early detection of subclinical maladaptive remodeling before observable structural changes on imaging, improve risk stratification for exercise-induced fibrosis, and may inform personalized training, which is essential for distinguishing detrimental cardiac remodeling from benign athletic heart adaptation (Che and Li, 2017; Goff, 2023; Lyu et al., 2024).

MicroRNAs (miRNAs) are conserved, single-stranded non-coding RNAs (ncRNAs) around 22 nucleotides in length that modulate post-transcriptional gene expression. Circulating miRNAs (c-miRNAs) exhibit altered expression in CVDs, and growing evidence supports their utility as clinical indicators. Both healthy and ill people exhibit exercise-induced alterations in c-miRNAs, with patterns that differ by exercise type, duration, and intensity (Wu et al., 2021; Saini et al., 2022).

This review seeks to: (1) examine the pathophysiology of exercise-induced myocardial injury; (2) evaluate the diagnostic efficacy of established biomarkers (cTn, BNP/NT-proBNP) and emerging biomarkers (Gal-3, ST2, Growth Differentiation Factor-15 (GDF-15), Heart-Type Fatty Acid-Binding Protein (H-FABP), Cardiac Myosin-Binding Protein C (cMyBP-C), and ncRNAs); (3) outline clinical applications for distinguishing physiological adaptation from pathological conditions, particularly in post-exercise chest pain assessment, return-to-play decisions, differentiation between athlete’s heart and cardiomyopathy, and monitoring exercise rehabilitation; and (4) identify current limitations, research deficiencies, and future directions, including sport-specific reference ranges, standardized protocols, multi-omics integration, and machine learning (ML) techniques to enhance personalized exercise prescription and monitoring in athletic populations.

This narrative review offers a thorough synthesis of information on exercise-related cardiac biomarkers, excluding quantitative meta-analysis due to variability in exercise regimens, biomarker assays, and study populations. A search in PubMed and Google Scholar (January 2000 to April 2026) used a mix of exercise-related keywords, including exercise-induced myocardial damage, cardiac biomarkers, hs-cTn, NPs, Gal-3, ncRNAs, and precision medicine. We included original research, systematic reviews, meta-analyses, and clinical recommendations that documented exercise-induced biomarker alterations in human participants. We included mechanistic animal studies only where human data were scarce, and all studies were published in English in peer-reviewed journals. We rejected case reports (except for unique clinical discoveries), conference papers, non-peer-reviewed sources, theoretical works, and research without original data. No formal quality assessment was conducted; nevertheless, we rigorously evaluated the sample size, follow-up duration, procedural standardization, and potential confounding variables, and we fully addressed the limitations throughout the publication. Findings were synthesized narratively by biomarker category, prioritizing large prospective studies and meta-analyses, but also including smaller research addressing particular inquiries. This narrative review, although conducted using systematic procedures, is susceptible to selection bias, which we addressed by frank reporting, incorporation of contradictory information, and recognition of gaps in the literature.

## 2 Pathophysiology of exercise-induced myocardial injury

### 2.1 Exercise-related cardiac stress and biomarker release

Moderate exercise enhances cardiovascular health, but excessive amounts of prolonged high-intensity endurance exercise may lead to cardiotoxic effects (Aizer et al., 2009; Myrstad et al., 2014; Mannakkara and Finocchiaro, 2023). Repeated acute stressors, mechanical strain on the heart, hypertensive responses, and transient inflammation lead to reversible elevations in cardiac biomarkers, including troponin (83% of athletes have abnormal hs-cTn T (hs-cTnT) levels post-exercise), BNP, and d-dimer (La Gerche et al., 2008; Sedaghat-Hamedani et al., 2015; Mannakkara and Finocchiaro, 2023). The benefits of exercise follow a U-shaped curve, with some cardiotoxicity at excessive levels (Mannakkara and Finocchiaro, 2023).

Importantly, the exercise-induced release of cardiovascular biomarkers (BNP and Cardiac Troponin (cTn)I/cTnT) represents a temporary physiological reaction indicative of reversible cardiac adaptation and increased membrane permeability, rather than irreversible cell death (Ranjbar et al., 2017; Donnellan and Phelan, 2018; López-López and Pareja-Galeano, 2018; Aengevaeren et al., 2021). The suggested mechanisms comprise: (1) augmented cardiomyocyte membrane permeability resulting in cytosolic leakage due to sarcolemma injury and oxidative stress from reactive oxygen species (ROS); (2) myocardial wall stress and distension, encompassing transient diastolic ventricular dysfunction and right ventricular (RV) predisposition to dilation; and (3) physiological structural adaptations characterized by enhanced membrane turnover and repair signaling through the Insulin-like Growth Factor-1/Phosphoinositide 3-kinase/Protein Kinase B (IGF-1/PI3K/Akt pathway) (Ranjbar et al., 2017; Donnellan and Phelan, 2018; López-López and Pareja-Galeano, 2018; Aengevaeren et al., 2021).

### 2.2 Oxidative stress, inflammation, and mitochondrial dysfunction

Intense exercise places a substantial strain on mitochondria, increasing cardiomyocyte oxygen demand by more than 10-fold (Shi et al., 2022). Increased cytoplasmic calcium, protein oxidation, and lipid peroxidation result from excess ROS generation overwhelming antioxidant defenses (such as superoxide dismutase (SOD)). Overdosing on calcium causes the mitochondrial permeability transition channel to open, releasing proapoptotic proteins and activating caspases, ultimately leading to cardiac apoptosis (Ascensão et al., 2005; Zhang et al., 2018; Li et al., 2021; Shi et al., 2022). Additionally, exercise promotes interstitial fibrosis and cardiomyocyte hypertrophy by inducing a proinflammatory response via NF-κB, thereby increasing TNF-α, IL-6, and IL-1β while reducing the anti-inflammatory IL-10 (Bernecker et al., 2013; Shi et al., 2022). Exercise-related cardiac biomarker release is caused by several interrelated processes, including oxidative stress, inflammation, calcium dysregulation, and mitochondrial dysfunction (Bernecker et al., 2013; Shi et al., 2022).

### 2.3 Clinical implications for biomarker interpretation

There is ongoing discussion over the possibility of cardiac injury from endurance exercise. During prolonged endurance sports (marathons, ultratriathlons), both professional and recreational athletes may suffer biomarker spikes; however, it is unclear whether these increases represent a normal physiological response or a clinically significant cardiac insult (Scharhag et al., 2008). Post-exercise cTnT, cTnI, and NT-proBNP are significantly elevated in children and adolescents, with 76% of individuals exceeding the URL for cTnT and 39% reaching AMI threshold levels (Cirer-Sastre et al., 2019). This emphasizes the importance of having reference ranges tailored to athletes and children.

Premature return to intense activity increases the likelihood of malignant arrhythmias and persistent inflammation, making myocarditis a unique clinical condition. The 2025 European Society of Cardiology Guidelines state that full symptom remission, normalized biomarkers and cardiac function, the absence of severe arrhythmias, and a repeat cardiac magnetic resonance (CMR) if edema or fibrosis were observed are necessary for a safe return to play (Bryde et al., 2023).

### 2.4 Exercise-induced cardioprotection

**Figure 1:**
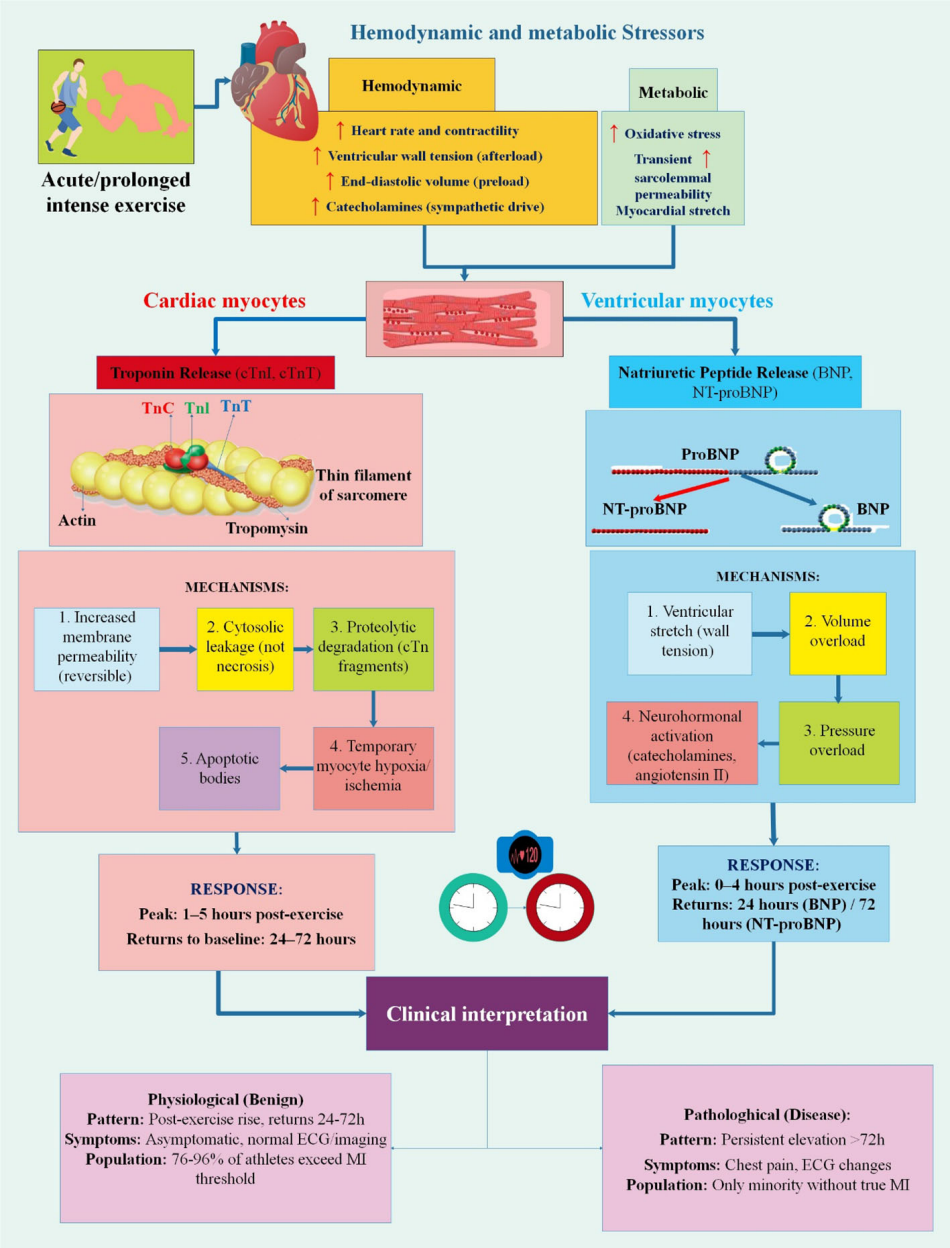
Comparative causes, release kinetics, and clinical interpretation of increases in NP (BNP, NT-proBNP) and cTn (cTnI, cTnT) after acute, intensive exercise. Different mechanisms are triggered by hemodynamic and metabolic stressors: reversible membrane permeability and cytosolic leakage from the sarcomere (troponin) in response to myocyte stretch, and neurohormonal activation (NPs). Peak time (cTn: 1-5h; NPs: 0-4h) and return to baseline (cTn: 24-72h; NPs: 24-72h) are two important differences. Serial sampling is necessary for clinical interpretation: pathogenic elevations persist for more than 72 hours, with increasing patterns and symptoms, whereas physiological (benign) elevations return to baseline within 24 to 72 hours, with normal ECG and imaging (Martinez et al., 2021; Cirer-Sastre et al., 2022; Omran et al., 2022; Shi et al., 2022).

Ironically, defensive systems are also induced by exercise. By reprogramming energy metabolism to induce trained immunity in macrophages, exercise-induced hypertrophic preconditioning reduces myocardial ischemia-induced damage, leading to reduced infarct size, improved cardiac function, and reduced myocardial fibrosis (Liu et al., 2025). Additionally, long-term exercise enhances endothelial function via increased NO generation and lowers chronic inflammation by downregulating TNF-α and IL-1β while activating anti-inflammatory mediators (IL-6, IL-1ra, and IL-10) (Chen et al., 2014; Kim and Baggish, 2017; Adams and Linke, 2019) (**Figure 1**).

## 3 Emerging established biomarkers (Gal-3, ST2, GDF-15, hs-CRP)

### 3.1 Galectin-3

Gal-3 is a beta-galactoside-binding lectin that mediates cardiac fibrosis in conditions such as moderate mitral stenosis by encouraging collagen deposition and fibroblast proliferation, which results in left atrial (LA) remodeling and stiffness. Impaired LA reservoir, conduit, and contractile performance results from elevated Gal-3 levels, which have a negative correlation with LA compliance, volume, and contractile function (measured by LA strain/speckle tracking). This remodeling reduces exercise tolerance by limiting the left atrium’s capacity to accommodate increased blood flow during physical activity. LA strain helps identify individuals with substantial functional restriction, and Gal-3 serves as a prognostic marker for assessing LA remodeling and the severity of dysfunction before symptom onset (Mahfouz et al., 2020; Arab et al., 2025).

In a study of 18 trained athletes, three cardiac biomarkers were evaluated before and immediately after a 60-kilometer ultramarathon: hs-cTnI (TnI, reflecting myocyte necrosis), NT-proBNP (reflecting myocyte stretch), and Gal-3 (reflecting cardiac fibrosis). All three biomarkers showed significant increases in post-marathon concentrations: median increases were 2.4-fold for Gal-3, 3.5-fold for NT-proBNP, and 3.3-fold for TnI. The frequency of results exceeding diagnostic limits increased considerably for NT-proBNP (0% *vs.* 28%, p=0.016) and Gal-3 (0% *vs.* 67%, p<0.001), whereas it did not change significantly for TnI (6% *vs.* 25%, p=0.15). The increases in the three biomarkers showed no discernible relationships. The researchers concluded that the heart’s structure and biology may be adversely affected by biochemical abnormalities associated with high-intensity endurance exercise (Salvagno et al., 2014).

In a study of 16 male recreational divers, Gal-3 and other cardiovascular biomarkers were evaluated before and after a prolonged non-dive interval and a 30-minute Self-Contained Underwater Breathing Apparatus (SCUBA) dive to a depth of 30 meters. At baseline and 30 minutes, 3 hours, and 6 hours after the dive, blood samples were taken. Gal-3, NT-proBNP, hs-TnI, myoglobin (Mb), and vascular endothelial growth factor (VEGF) were significantly increased immediately after diving, according to the results. While NT-proBNP and hs-TnI continued to rise during recovery, Gal-3 and Mb recovered to baseline levels. After three hours, VEGF levels dropped dramatically, and by six hours, they had recovered to baseline. During recovery, endothelin-1 initially decreased, then increased, without returning to baseline. Although it is not yet known whether repeated dives have positive or negative long-term cardiovascular effects, the authors concluded that these biomarker changes reflect a complete but transient adaptation of the circulatory and muscular systems to SCUBA diving conditions (Žarak et al., 2020).

By promoting collagen deposition and fibroblast proliferation, Gal-3 drives heart fibrosis. Exercise tolerance is reduced by elevated Gal-3, which is negatively correlated with contractile function and LA compliance (Mahfouz et al., 2020; Arab et al., 2025). According to recent research, skeletal muscle, not only cardiac tissue, may produce Gal-3 during exercise (Le Goff et al., 2020).

Clinical Utility: Before symptom onset, Gal-3 predicts LA remodeling and the severity of dysfunction (Mahfouz et al., 2020; Arab et al., 2025). When used with BNP and ST2, it improves risk stratification.

### 3.2 Suppression of tumorigenicity 2

ST2 increases significantly during ultratrail runs or marathons. In contrast to Gal-3, ST2 often rises rather than falls throughout recovery, sometimes exceeding the clinical cutoff (35 ng/dL) across multiple time points (Le Goff et al., 2020). The existing information on ST2 levels after exercise in healthy individuals was assessed in a thorough review. Up to October 2020, a search of PubMed, ISI Web of Science, and Scopus yielded 6 studies with 349 participants (73% male). ST2 levels increase during exercise, according to most studies. A clinical cut-off of 35 ng/dL for ST2 was reported in three investigations involving 219 people; after running, 92.7% of subjects had ST2 levels above this cut-off. According to the authors, most studies report higher ST2 levels during exercise, with a notable proportion exceeding the 35 ng/dL threshold. However, the small number of patients examined, the scarcity of imaging data, and the absence of long-term follow-up call for additional prospective, larger studies (Martins da Costa et al., 2021).

Although it may lack disease specificity and is not often used for routine diagnosis, the 2013 American Association for Clinical Chemistry/American Heart Association (AHA) guidelines endorse ST2 (soluble IL-1 receptor-like) for additive risk stratification in HF (Goff, 2023). ST2 reflects inflammation, tissue fibrosis, matrix remodeling, and myocyte strain. Endurance athletes may have a greater incidence of cardiac fibrosis, which may contribute to arrhythmias and sudden cardiac death (SCD); intense exercise induces multi-organ stress with increases in cardiac and inflammatory biomarkers. Although biomarker release may be temporary and reversible, it remains unclear whether prolonged, frequent high-intensity exercise (HES) results in HF and long-term fibrosis (Goff, 2023). While high Gal-3 and ST2 levels are risk factors for both HF patients and the general population, the author notes that a new study shows that regular HES increases these biomarkers, which may help identify athletes at risk for SCD beyond existing biomarkers. The paucity of long-term follow-up data, the inability to identify the primary sources of circulating ST2 in healthy persons, and the lack of knowledge about the predictive ability of acute exercise-induced elevations for chronic maladaptive remodeling are significant gaps. Additional prospective studies with long-term outcome monitoring, CMR imaging for fibrosis, and serial biomarker assessments are needed to determine clinical significance (Goff, 2023).

### 3.3 Growth Differentiation Factor-15

GDF-15 is a stress-responsive cytokine. Acute HES (marathons) results in a short-lived but significant increase in circulating GDF-15 that persists for many hours after recovery. Consistent ET, however, may ultimately result in lower baseline GDF-15 levels, which are linked to decreased fat mass and enhanced metabolic flexibility (Zhang et al., 2019; Campderrós et al., 2020; Conte et al., 2020; Laurens et al., 2020).

Recurrent MI and death risk are linked to GDF-15. Over 7.75 years, patients with GDF-15 and NT-proBNP levels above the median had less than 10% event-free survival (Kempf et al., 2006). After acute coronary syndrome, a roughly twofold greater risk of death is associated with high GDF-15 (Liu et al., 2019). Following Ischemia/Reperfusion (I/R), GDF-15 knockout animals exhibited much bigger infarct volumes, indicating protective effects via the PI3K/Akt pathway (Kempf et al., 2006; Khan et al., 2009; Bonaca et al., 2011; Schaub et al., 2012; Liu et al., 2019; Laurens et al., 2020).

For cardiovascular risk assessment, GDF-15 is more useful than troponin or NT-proBNP. Significant decreases in fat mass and improved insulin sensitivity are associated with exercise-induced increases in GDF-15 in obese people (Zhang et al., 2019; Campderrós et al., 2020; Conte et al., 2020; Laurens et al., 2020). GDF-15 is being investigated as a potential therapeutic target for cachexia, HF, and metabolic syndrome (Laurens et al., 2020; Pereira et al., 2026).

### 3.4 High-sensitivity C-reactive protein

Regular exercise significantly reduces hs-CRP, an important inflammatory biomarker associated with CVD risk. Since elevated hs-CRP (>2 mg/L) indicates increased risk, engaging in at least 150 minutes of moderate-intensity or 75 minutes of vigorous-intensity exercise each week reduces systemic inflammation and cardiovascular events. Compared with a sedentary lifestyle, both moderate and intense exercise are associated with lower hs-CRP levels; in individuals with heart disease, regular exercise may reduce hs-CRP by 15–40%. A potent biomarker for primary and secondary prevention, hs-CRP is often used alongside cholesterol screening to assess inflammatory risk. Guidelines include quitting smoking, combining exercise with a healthy diet (such as a Mediterranean diet), and aiming for 150 minutes of moderate or 75 minutes of strenuous activity per week for those at intermediate cardiovascular risk. Additionally, they need to speak with a medical expert about hs-CRP testing (Musunuru et al., 2008; Bergström et al., 2012; Hammonds et al., 2016; Ridker, 2016). Regular exercise may lower hs-CRP by 15–40% in those with heart disease.

The impact of ET on cardiovascular risk factors and the progression of abdominal aortic aneurysms (AAAs) was evaluated in a meta-analysis of 8 Randomized Controlled Trials (RCTs) including 588 AAA patients. Exercise significantly improved hs-CRP (Standardized Mean Difference (SMD): -0.56 mg/dL), peak oxygen consumption (V̇O_2peak_, SMD: 0.4 mL/kg/min), triglycerides (SMD: -0.39 mg/dL), and anaerobic threshold (SMD: 0.75 mL/kg/min). AAA diameter, total cholesterol, high-density lipoprotein (HDL)/low-density lipoprotein (LDL) ratio, HDL, LDL, and matrix metalloproteinase (MMP)-9 were not substantially impacted by exercise. The researchers concluded that an exercise intervention improves cardiorespiratory capacity and several inflammatory and metabolic markers in patients with AAA. Still, it does not significantly reduce AAA size or improve the overall lipid profile. Exercise might be used as a supplemental strategy to improve health and reduce the risk of AAA formation (Han et al., 2024) (**Table 1**).

**Table 1:** Overview of principal studies investigating exercise-related CVD biomarkers.

Biomarker	Study population	Exercise protocol	Sampling time	Main findings	Clinical implications	Limitations	Ref.
hs-cTnI, hs-cTnT	97 recreational cyclists	91-km race	Pre-race; 0h, 3h, 24h post-race	76–87% of hs-cTnI and 95–96% of hs-cTnT fulfilled MI criteria at 3 hours. hs-cTnT peaked instantly, whereas hs-cTnI peaked at 3 hours.	The current MI criteria do a poor job of distinguishing exercise-induced increases from acute MI.	Single race type; no long-term follow-up; no cardiac imaging to exclude subclinical disease	(Skadberg et al., 2018)
hs-cTnT	733 children/adolescents (12.2 ± 1.7y; 40% female)	20-m shuttle run test	Rest and 3h post-exercise	After exercise, 56.2% had elevated hs-cTnT levels. Just 7.5% of URLs exceeded. The growth was somewhat greater for girls.	There is substantial variation in response that is not well explained by conventional parameters.	No serial sampling; single time point post-exercise; no imaging	(Conesa-Milian et al., 2026)
hs-cTnT, hs-cTnI	59 middle-aged/older male athletes (with/without coronary atherosclerosis)	Thorough cycling test	Serial measurements	Both troponins increased (1.55x and 2.76x). No differences in elevation patterns among severe stenosis, high coronary artery calcium score, and controls.	Coronary atherosclerosis does not worsen exercise-induced cTn release.	All male; single exercise session; moderate sample size	(Janssen et al., 2024)
hs-cTnT	11 sedentary healthy males	40 min treadmill: intermittent (50–80% HRR) *vs.* continuous (60% HRR), crossover	Before, during, 1h post	hs-cTnT increased post-exercise with no difference between protocols. Continuous exercise produced higher hs-cTnT than intermittent. Creatine Kinase-Myocardial Band (CK-MB) unchanged.	Continuous activity produces greater cTnT than intermittent exercise; permanent cardiomyocyte death is improbable.	Very small sample (n=11); single session; no long-term outcomes	(Ranjbar et al., 2017)
cTnI	15 Revasc-Coronary Artery Disease (CAD) *vs.* 22 Wo-CAD	High-intensity spinning (20, 40, 60 min)	Serial (including 3h and 24h)	cTnI peaked at 60 min and 3h. Revasc-CAD showed no decrease between 3 and 24h after maximal exercise. One case of in-stent stenosis showed an increase in cTnI.	Evolving coronary artery blockage may be detected by a persistent rise in cTnI after maximal exercise.	Small CAD subgroup; single center; one case limits generalizability	(Hove et al., 2026)
cTnI	25 children with chest pain during physical activity	Retrospective, exercise history	Serial over 48h	cTnI increased to 133 ng/L post-exercise, decreased to 3.25 ng/L at 48h. Negatively correlated with age; positively correlated with exercise duration.	Exercise-related increases should be considered before diagnosing heart disease in children.	Retrospective design; no control group; variable exercise types	(Ates et al., 2025)
cTnI (saliva *vs.* serum)	82 male runners (54 exercise, 28 control)	5-km time-trial *vs.* non-exercise control	Pre, early post, 4h, 24h	Salivary and serum cTnI showed proportional agreement, with parallel increases that peaked at 4 hours (0.62 *vs.* 0.76 ng/mL) and returned to baseline by 24 hours.	Salivary cTnI with POC assay may be a non-invasive alternative to blood draws.	Male only; single race distance; POC device-specific validation needed	(Ovchinnikov, 2025)
BNP	340 consecutive HF patients (EF<45%)	3-month ET	Pre- and post-training	High-BNP group (≥200 pg/mL): A 8.3% increase in peak V̇O_2_. The top tertile of improvement (≥13.0%) had lower 46-month mortality/HF hospitalization than the bottom tertile (37.9% *vs.* 54.4%).	ET improves exercise capacity even in advanced HF with high BNP; greater improvement predicts better outcomes.	Non-randomized; single center; baseline differences between groups	(Nakanishi et al., 2017)
NT-proBNP	40 asymptomatic hypertensive patients	ECG treadmill test	Before and after exercise	No correlation between NT-proBNP and E/E’ ratio pre- or post-exercise.	E/E’ is not a reliable marker of exercise-induced LV dysfunction in asymptomatic hypertension.	Small sample; single center; no validation cohort	(Paun et al., 2021)
NT-proBNP	90 cyclists	210–250 km ride	Post-ride	Exercise-Associated Hyponatremia (EAH) occurred in 4.5% and was associated with weight gain (3.4 kg). EAH cyclists had higher post-exercise NT-proBNP than eunatremic controls.	EAH is linked to subclinical ventricular strain; overconsumption of hypotonic fluids is the main cause.	Observational; no baseline NP levels; no long-term follow-up	(Harris et al., 2012)
Gal-3, TnI, NT-proBNP	18 trained athletes	60-km ultramarathon	Pre- and post-race	Increases: TnI 3.3×, NT-proBNP 3.5×, Gal-3 2.4×. % over diagnostic limits: TnI 25%, NT-proBNP 28%, Gal-3 67% (p<0.001 for Gal-3). No correlations among biomarkers.	High-intensity endurance exercise produces biochemical anomalies that may affect cardiac structure.	Very small sample (n=18); no imaging; no long-term follow-up	(Salvagno et al., 2014)
Gal-3, NT-proBNP, hs-TnI, Mb, VEGF	16 male recreational divers	30-min SCUBA dive to 30 m	Baseline; 30 min, 3h, 6h post-dive	All biomarkers increased immediately post-dive. Gal-3 and Mb returned to baseline; NT-proBNP and hs-TnI continued to rise during recovery.	Biomarker changes represent temporary adaptation; long-term effects of repeated dives are unknown.	Small sample; all male; single dive; no long-term outcomes	(Žarak et al., 2020)
Gal-3, ST2	19 marathon, 27 ultratrail, 14 control runners	Marathon (42 km), ultratrail (67 km), control (10 km)	Pre (T0), immediate post (T1), 3h post (T2)	All biomarkers rose from T0 to T1. By T2, Gal-3 had normalized, but ST2 continued to increase—occasionally surpassing clinical cutoffs. Biomarker kinetics varied significantly by run type.	ST2 and Gal-3 reflect different recovery kinetics; ST2 elevation persists longer.	No cardiac imaging; single recovery time point; no long-term fibrosis assessment	(Le Goff et al., 2020)
ST2	Systematic review: 6 studies, 349 participants (73% male)	Various running protocols	Various (pre, post, recovery)	ST2 increases with exercise in most studies. In 3 studies (n=219), 92.7% had ST2 >35 ng/dL (clinical cutoff) after running.	ST2 frequently exceeds clinical cutoffs in healthy athletes; thresholds may need adjustment.	Limited number of studies; no imaging; no long-term follow-up; heterogeneity of protocols	(Martins da Costa et al., 2021)
GDF-15	Review (marathon runners, CAD, HF, knockout mice)	Various (marathon, I/R models)	Various	GDF-15 rises with acute exercise but falls with chronic training. GDF-15 knockout mice show larger infarcts after I/R. In CAD patients, GDF-15 correlates with the Gensini score.	GDF-15 has both protective (acute) and risk-associated (chronic) roles; it is a potential therapeutic target.	Heterogeneous studies; mostly observational; causal relationships not established	(Kempf et al., 2006; Khan et al., 2009; Bonaca et al., 2011; Schaub et al., 2012; Liu et al., 2019; Zhang et al., 2019; Campderrós et al., 2020; Conte et al., 2020; Laurens et al., 2020; Pereira et al., 2026)
hs-CRP, oxidative stress, cortisol	Sleep deprivation (SD) + low- *vs.* HES	Low-intensity exercise (LES) *vs.* HES aerobic exercise (AE) after 3 days SD	Before and after exercise	SD elevated cortisol, hs-CRP, and oxidative stress. LES lowered cortisol and oxidative stress, but HES exacerbated both. hs-CRP remained unchanged with either exercise intensity.	LES is safe and appropriate for SD individuals; HES may worsen stress responses.	Short SD duration (3 days); no polysomnography; single exercise session	(Park et al., 2023)
hs-CRP, lipids, V̇O_2peak_	Meta-analysis: 8 RCTs, 588 AAA patients	Various ET programs	Pre- and post-training	Exercise improved hs-CRP (SMD -0.56), V̇O_2peak_ (SMD 0.4), triglycerides (SMD -0.39), and anaerobic threshold (SMD 0.75). No change in AAA diameter or most lipids.	Exercise improves cardiorespiratory and inflammatory markers in AAA but does not reduce aneurysm size.	Heterogeneous protocols; moderate quality evidence; no long-term AAA outcome data	(Han et al., 2024)
BNP/NT-proBNP, inflammatory markers	Meta-analysis: 9 RCTs, HF patients	HIIT *vs.* MCT *vs.* usual care	Pre- and post-training	HIIT significantly reduced BNP/NT-proBNP (SMD -1.33). No significant effect on CRP, TNF-α, and IL-6. Some studies have reported paradoxical increases in IL-6 with HIIT.	HIIT may be more effective than MCT for reducing NP in HF; the effects on inflammatory markers are unclear.	Heterogeneous protocols; small number of RCTs for some outcomes; bias risk in some studies	(Zhang et al., 2025)

### 3.5 Summary of evidence gaps and limitations for other biomarkers (Gal-3, ST2, GDF-15, hs-CRP)

Exercise-related biomarkers such as Gal-3, ST2, GDF-15, and hs-CRP have received little research attention. Direct comparisons are challenging due to methodological limitations in the studies, including small sample sizes (16–219 participants), brief follow-up periods, and varied exercise regimens. Since the long-term clinical implications are yet unclear, a significant information vacuum exists about whether recurring training-induced biomarker spikes suggest chronic heart fibrosis or potential unfavorable cardiovascular events. Given that healthy athletes are often above clinical diagnostic cutoffs (92.7% for ST2, 67% for Gal-3), there are serious concerns about the suitability of applying thresholds obtained from HF patients to physically active groups. Inconsistent outcomes, such as contradictory findings on BNP responses between moderate continuous training (MCT) and high-intensity interval training (HIIT), further show methodological variation. Whether circulating ST2 and Gal-3 during exercise originate from cardiac tissue or skeletal muscle, and whether brief elevations are indeed harmless, remain unanswered questions. To clarify the clinical importance of these biomarkers, future research should concentrate on large-scale prospective studies with extended follow-up periods, include CMR imaging, develop age- and sport-specific reference ranges, and use multi-omics approaches.

## 4 Emerging novel biomarkers

Differential diagnosis is difficult because troponin elevation implies myocardial damage but does not identify the specific mechanism involved (increased membrane permeability, apoptosis, or temporary ischemia). This is especially challenging in athletes, in whom strenuous activity causes well-documented, transient troponin elevations (Lyngbakken et al., 2019; Celeski et al., 2024). Exosomes (EXOs) and cytokines are being studied in relation to exercise response and CVD diagnosis. Because of their stability and packaging potential, EXOs have therapeutic, prognostic, and diagnostic applications; cytokines, on the other hand, may predict CV risk and improve training methods (Lyngbakken et al., 2019; Haller et al., 2023; Peng and Zhang, 2024).

The main emphasis is on early detection of subclinical injury from intense exercise, cardiac fibrosis, and structural remodeling. In endurance athletes, Gal-3 and ST2 detect early remodeling. Enzymes, chaperones, and cytokines help distinguish pathological remodeling from common “athlete’s heart” disorders. More than 200 inflammation-related plasma proteins associated with cardiac changes during intense exercise have been identified by proteomics (Lyngbakken et al., 2019; Haller et al., 2023; Peng and Zhang, 2024).

### 4.1 Heart-type fatty acid-binding protein

#### 4.1.1 Release kinetics

In contrast to skeletal muscle (the diaphragm has 25% of cardiac levels), H-FABP, which *FABP3* encodes, is significantly expressed in the heart (ventricles 0.46 mg/g, atria 0.25 mg/g) (Varrone et al., 2013; Rezar et al., 2020). H-FABP is rapidly produced after myocardial injury due to its small size and cytoplasmic localization; it may be detected as early as 15 minutes after iatrogenic infarction, earlier than troponins. Twenty hours after the beginning of AMI, it is removed by the kidneys and returns to baseline (Zoair et al., 2015; Ponikowski et al., 2016; Ye et al., 2018; Rezar et al., 2020). In athletes, low-intensity exercise (LES) produces insignificant changes in H-FABP, but HES induces noticeable short-term increases (peaking 3–4 hours after exercise). It may be possible to differentiate between skeletal and cardiac muscle damage using the Mb-to-H-FABP ratio (Thomas and Motley, 1984; Mehdikhani, 2010; Fortunato et al., 2018).

#### 4.1.2 Diagnostic interpretation

In HF, elevated H-FABP levels correlate with NYHA class and are inversely associated with Ejection Fraction (EF), independently predicting worse outcomes. Combining H-FABP with BNP enhances risk classification as they indicate distinct processes (strain *vs.* chronic injury) (Zoair et al., 2015; Ponikowski et al., 2016; Ye et al., 2018; Rezar et al., 2020). Researchers evaluated the relationship between stable Coronary Artery Disease (CAD) and changes in H-FABP levels before and after exercise stress testing (EST) in an observational study of 47 participants (26 with substantial coronary lesions >70% stenosis, 21 with normal coronary architecture). Contrary to expectations, H-FABP levels tended to decrease from baseline at 3 hours rather than rise during EST. Both groups showed a statistically significant decrease (p<0.05); the CAD group experienced a greater reduction (2.79 ± 2.57 ng/mL) than the control group (0.84 ± 2.07 ng/mL; p=0.009). The researchers concluded that, rather than increasing as an indicator of myocardial injury, H-FABP levels decrease during EST, most likely due to exercise-induced proteinuria (Arı et al., 2011).

#### 4.1.3 Clinical utility

A prospective observational study examined the effects of 8 months of regular physical activity on biomarkers of myocyte ischemia (H-FABP), matrix remodeling/vascular stress (sST2), and inflammation (suPAR) in 98 participants who completed the exercise program. There was no discernible change in suPAR levels, a significant drop in H-FABP from 1.86 to 1.29 ng/mL (p<0.01), and an increase in sST2 from 6,126 to 6,919 pg/mL (p=0.045). While the increase in sST2 may suggest physiological, exercise-induced vascular stress, the authors interpret the decrease in H-FABP as evidence of improved perfusion and reduced subclinical myocardial ischemia, perhaps due to more effective metabolism and electrolyte balance. The effects of regular exercise on these biomarkers in individuals with ischemic cardiomyopathy should be investigated further (Trial registration: NCT02097199) (Sponder et al., 2019).

#### 4.1.4 Summary of evidence gaps and limitations for H-FABP

There are several limitations to research on H-FABP as a cardiovascular and exercise biomarker, including inconsistent findings. One study surprisingly reported a reduction after EST due to proteinuria, a significant confounding factor, although most studies show that H-FABP rises after myocardial injury or intense exercise. The narrow diagnostic window (return to baseline within 20 hours) limits clinical value. The majority of studies have small sample sizes (n=47–98) and lack external validation. The inability to distinguish between the cardiac and skeletal muscle origins of H-FABP during exercise is a significant gap. In terms of predictive value for HF and CAD, H-FABP has not surpassed hs-cTn, and no guidelines recommend its routine use. Standardized procedures, larger multicenter studies, direct comparisons with established biomarkers, and validated cutoffs to differentiate pathological from normal increases are all necessary for future studies.

### 4.2 Cardiac myosin-binding protein C

#### 4.2.1 Release kinetics and physiology

cMyBP-C is encoded by the *MYBPC3* gene and is a 140-kDa protein composed of three fibronectin type III domains (C0–C10) and eight immunoglobulin-like domains. Between C1 and C2 lies a regulatory M-domain with phosphorylation sites essential for regulating cardiac contractility. By binding to myosin subfragments S1 and S2, cMyBP-C, which is localized to the sarcomere C-zone, limits myosin head movement and cross-bridge formation by promoting the super-relaxed (SRX) state via the interacting heads motif (IHM) (Greenman et al., 2026). Additionally, it binds to actin and shifts tropomyosin, exposing calcium-independent myosin-binding sites, thereby increasing thin-filament sensitivity to calcium and promoting cross-bridge formation at lower calcium concentrations. Myofilament calcium sensitivity decreases, and crossbridge dynamics during contraction and relaxation accelerate when functional cMyBP-C is lost (Greenman et al., 2026). In conclusion, cMyBP-C modulates sarcomere activity by activating the thin filament and acting as a brake on the thick filament (promoting the non-crossbridge IHM/SRX conformation). It is still unknown how and when these interactions occur *in vivo*, even though phosphorylation and other post-translational modifications (PTMs) appear to be significant regulators (Greenman et al., 2026). cMyBP-C is a rapid-release biomarker of subclinical myocardial injury because it rises earlier and clears faster than cTn. Faster cross-bridge cycling and increased contraction velocity are made possible by cMyBP-C phosphorylation during adrenergic stimulation, such as exercise (Mamidi et al., 2014; Thoonen et al., 2015; Tong et al., 2017; Riveland et al., 2025).

#### 4.2.2 Diagnostic interpretation

The thick filament protein cMyBP-C is specific to the heart muscle. Exercise stress increased serum cMyBP-C in all 158 individuals (75 men, 83 women) who underwent exercise stress echocardiography with pre- and post-stress blood collection and were monitored for 1–1.5 years. There were 7 critical events (death, MI, stroke, or pulmonary embolism) and 27 major events (death, MI, revascularization, invasive cardiovascular treatments, or cardiovascular hospitalization). After correction for cardiovascular risk factors and sex, a pre-stress cMyBP-C threshold with 96% sensitivity for major events yielded a hazard ratio (HR) of 8.1 (p=0.041). On echocardiography, the majority of participants with critical events (6 out of 7) had normal EF. For critical events, pre-stress cMyBP-C showed an area under the curve (AUC) of 0.91 and an HR of 13.8 (p=0.000472) (Tong et al., 2017). The researchers determined that baseline cMyBP-C levels correlate with several cardiovascular disorders and may serve as a screening biomarker for severe CVD owing to their elevated sensitivity (Tong et al., 2017).

cMyBP-C is an emerging biomarker of myocardial damage that rises earlier and clears more quickly than cTn, with comparable diagnostic power for myocardial infarction and prognostic utility in acute HF. However, its relationship with troponins in patients with chronic HF (CHF) undergoing exercise remains unclear. In a *post hoc* analysis of the SMARTEX study, 205 symptomatic CHF patients with reduced EF (CHFrEF) were randomly assigned to either 12 weeks of HIIT or MCT [combined as the intervention group (IG)] or to a control group (CG) that received exercise guidelines. There were no notable variations in cMyBP-C level changes across groups (ΔcMyBP-C IG: -0.5 *vs.* CG: -0.7). A substantial correlation was observed between changes in log cMyBP-C and changes in log hs-cTnI (R = 0.52, 95% Confidence Interval (CI) 0.37-0.66, P<0.001); hs-cTnI increased by approximately 5% for every 10% rise in cMyBP-C (Riveland et al., 2025). The authors concluded that in patients with CHFrEF, increases in cMyBP-C are strongly correlated with hs-cTnI levels during structured ET, suggesting that cMyBP-C may eventually serve as a marker of subclinical myocardial injury (Riveland et al., 2025).

#### 4.2.3 Clinical utility

cMyBP-C is a novel biomarker of myocardial injury that elevates sooner and diminishes more rapidly than cTn, exhibiting similar diagnostic efficacy for myocardial infarction and prognostic value in acute HF (Mamidi et al., 2014; Thoonen et al., 2015; Tong et al., 2017; Riveland et al., 2025).

#### 4.2.4 Summary of evidence gaps and limitations for cMyBP-C

Investigations into cMyBP-C as a cardiovascular biomarker show potential but have significant limitations. The prospective study (n=158) had strong predictive value (AUC 0.91) but was limited by a small sample size, a brief follow-up period (1–1.5 years), and a lack of external validation. The majority of subjects experiencing critical episodes (6 out of 7) had normal EFs, without any comparison to CMR imaging. The SMARTEX *post hoc* analysis (n=205) revealed a moderate association (R = 0.52) between cMyBP-C and hs-cTnI; nevertheless, conclusions about the effects of exercise intensity were constrained by the amalgamation of IGs. Significant unresolved concerns include: the lack of standardized tests and cutoffs, challenges in distinguishing physiological from pathological release, insufficient direct comparisons with hs-cTn in athletes, and ambiguity regarding cardiac vs skeletal muscle specificity. Future investigations need extensive multicenter trials, sport-specific reference ranges, extended follow-up, and validation for hypertrophic cardiomyopathy screening in athletes.

### 4.3 Cardiac-specific non-coding RNAs

#### 4.3.1 Release kinetics and mechanisms

NcRNAs, such as miRNAs and long ncRNAs (lncRNAs), have recently been investigated for their exercise-associated cardioprotective effects, as they play essential roles in regulating gene expression during cardiovascular adaptation to physical activity (Yuan et al., 2025). Important cellular processes, including angiogenesis, apoptosis, fibrosis, and hypertrophy, are mediated by these ncRNAs. For instance, lncRNAs regulate gene expression networks that affect cardiovascular health, while miRNAs such as miR-1, miR-133, and miR-206 modulate cardiac remodeling and function in response to exercise. Epigenetic alterations, including DNA methylation and histone modifications, also mediate the cardioprotective benefits of exercise. Exercise alters DNA methylation patterns in genes related to inflammation, oxidative stress, and metabolic control, and increases histone acetylation and methylation changes linked to cardioprotective gene programs (Yuan et al., 2025). These epigenetic processes elucidate the enduring benefits of exercise and illustrate how it may alter gene expression in both heritable and reversible ways (Yuan et al., 2025). Acute exercise swiftly mobilizes c-miRNAs, including miR-21, miR-146a, miR-221, and miR-222, in response to tissue stress, but chronic exercise alters specific sets, such as miR-139, miR-143, miR-223, and miR-330 (Ultimo et al., 2018).

From an evolutionary standpoint, lncRNAs associated with the heart and regulating metabolism could be more conserved across species than those associated with brain processes (Kay and Soltani, 2021; Metzinger and Metzinger-Le Meuth, 2026). LncRNAs functioning inside core metabolic pathways may be subject to more stringent evolutionary constraints since these pathways are old and well conserved across eukaryotes. Neuro-specific lncRNAs, on the other hand, may change more quickly, as they are often linked to cellular diversity, lineage-adaptive features, and rapid regulatory innovation. This distinction has significant implications for biomarker development, as discussed by Metzinger and Metzinger-Le Meuth (Metzinger and Metzinger-Le Meuth, 2026): lineage-specific lncRNAs might require species-matched experimental validation, whereas conserved metabolism-related lncRNAs might be more reliably translated from animal models to human populations. Biomarker functional prioritization, experimental modeling, and ultimately therapeutic targeting are all significantly impacted by knowing which lncRNAs are conserved, which are lineage-specific, and which molecular characteristics, such as secondary structure, genomic context, and protein-binding domains, are maintained across species (Metzinger and Metzinger-Le Meuth, 2026).

In a study of 20 patients with CAD (10 male, 10 female), researchers evaluated miRNA expression in response to maximal ergospirometry. Blood samples were obtained before and five minutes after maximal cycle ergospirometry, and qRT-PCR was used to analyze 187 target miRNAs. The expression levels of 33 miRNAs were substantially altered during exercise, with 16 of these miRNAs showing significant sex differences. Nine miRNAs (let-7e-5p, miR-1, miR-19b-1-5p, miR-103a-3p, miR-148b-3p, miR-181b-5p, miR-188-5p, miR-423-5p, and miR-874-3p) with statistically different responses to exercise across sexes were identified using multivariate analysis. These miRNAs are linked to oxidative stress, angiogenesis, and glucose metabolism pathways (Mayr et al., 2021). The researchers determined that miRNA expression in CAD patients differs by sex and is responsive to maximum ergospirometry, indicating that disease-specific miRNA expression during maximal exercise may act as a future prognostic indicator of patient outcomes (Mayr et al., 2021).

#### 4.3.2 Diagnostic interpretation

In 20 previously sedentary individuals from the Health, Risk Factors, ET and Genetics (HERITAGE) Family Study, researchers examined how 20 weeks of endurance ET affected c-miRNA expression. Nine miRNAs were downregulated (miR-486-5p, let-7b-5p, miR-29c-3p, let-7e-5p, miR-93-5p, miR-7-5p, miR-25-3p, miR-92a-3p, and miR-29b-3p; fold change 0.64–0.83, p=0.0002–0.01), while five miRNAs were upregulated (miR-142-3p, miR-221-3p, miR-126-3p, miR-146a-5p, and miR-27b-3p). These 14 miRNAs target genes implicated in more than 345 biological pathways, according to enrichment analysis (Barber et al., 2019). The authors provide that these data demonstrate that regular exercise significantly alters the c-miRNA profile (Barber et al., 2019).

To examine acute exercise responses of c-miRNAs (identified as biomarkers of heart disease), blood samples were collected at baseline and at 0, 24, and 72 hours after 10-kilometer, half-marathon, and marathon races in healthy, active individuals. Despite the absence of symptoms or indicators of heart damage, all nine cardiac biomarkers (hs-cTnT, NT-proBNP, etc.) rose in a dose-dependent manner with exercise duration. Five c-miRNAs were dysregulated after a 10-kilometer run, and 19 c-miRNAs after a marathon. Each race induced distinct qualitative and quantitative changes in cardiac adaptation-associated miRNAs. Notably, “pseudo-disease” signatures were also observed, and several c-miRNAs previously associated with heart disease were either undetectable or stable during exercise (indicating their discriminative potential) (de Gonzalo-Calvo et al., 2018). The researchers determined that clinicians must recognize that exercise-induced “pseudo-disease” signatures can influence the interpretation of miRNA data; however, c-miRNAs may provide supplementary diagnostic value alongside established biomarkers such as hs-cTnT or NT-proBNP in patients with cardiac dysfunction symptoms following endurance exercise (de Gonzalo-Calvo et al., 2018).

#### 4.3.3 Clinical utility

MiR-1, miR-133a, and miR-206 link with VO_2max_ in marathon runners. Long-term endurance training lowers cardiac miR-208a expression (increasing contractile efficiency) but enhances miR-92a and miR-92b in CAD patients (Ultimo et al., 2018). Exosomal miRNAs are dynamically changed by exercise: HIIT raises circulating exosomal miR-133a 2–3 fold, targeting CTGF and decreasing cardiac fibrosis (Yu et al., 2026).

Using an apolipoprotein E (ApoE)^-/-^ mouse model, this study examined whether aerobic exercise (AE) stabilizes atherosclerotic plaques via vascular smooth muscle cell (VSMC) miRNA reprogramming, focusing on the miR-15a-5p/*Sema3A* axis. ApoE^-/-^ mice fed a high-fat diet were assigned to either a sedentary or moderate-intensity treadmill training regimen for 12 weeks. Exercise inhibited macrophage infiltration, increased collagen content, decreased lipid content, and reduced plaque vulnerability. Exercise substantially lowered miR-15a-5p, which was significantly elevated in atherosclerotic aortas, according to integrative miRNA profiling. The plaque vulnerability score and miR-15a-5p levels were positively correlated in human carotid plaques. Mechanistically, miR-15a-5p repressed *Sema3A* expression by directly targeting its 3’-UTR. *In vivo* overexpression of VSMC-specific miR-15a-5p destabilized plaques, accelerated phenotypic switching, and downregulated contractile markers (Hu et al., 2026). The researchers revealed that AE stabilizes plaques, relieves *Sema3A* repression, and maintains the contractile VSMC phenotype via downregulating miR-15a-5p. Its potential as a therapeutic target for atherosclerosis is supported by the association between vulnerability and high miR-15a-5p levels in human plaques (Hu et al., 2026).

In a rat model of CHF induced by left anterior descending coronary artery ligation, researchers examined the effects of AE. Starting four weeks after surgery, CHF rats underwent eight weeks of AE training. Exercise reduced ROS and inflammatory cytokines (TNF-α, IL-6, IL-1β), decreased MMPs (MMP-2, MMP-9), reduced myocardial apoptosis, and increased autophagy-related proteins (beclin-1, LC3B-II). It also enhanced cardiac function and left ventricular remodeling. All the positive effects of exercise were abolished when the lncRNA metastasis-associated lung adenocarcinoma transcript 1 (*MALAT1*) was overexpressed. Mechanistic studies showed that *MALAT1* overexpression promoted apoptosis and decreased autophagy by downregulating miR-150-5p and suppressing the PI3K/Akt signaling pathway. Using a mimic to restore miR-150-5p reduced apoptosis, increased autophagy, and reactivated PI3K/Akt (Hu et al., 2021). The researchers determined that the miR-150-5p/PI3K/Akt signaling pathway mediates these protective effects, and that AE improves heart function in CHF by inhibiting *MALAT1* (Hu et al., 2021). HF and cardiac hypertrophy are greatly influenced by several circRNAs and lncRNAs. Bvht regulates cardiac growth via *Mesp1* (which has no human counterpart), while *Mhrt* binds to *Brg1* to prevent abnormal hypertrophy. Patients with hypertension have altered expression of *Carmen*, *Fendrr*, and *Mhrt*; *Carmen* is positively correlated with left ventricular mass index, whereas *Fendrr* and *Mhrt* are negatively correlated with hypertrophy. By blocking autophagy and altering epigenetic modifications, *Chast* and *Chaer* promote pathological hypertrophy. As competitive endogenous RNAs (ceRNAs), *Apf* and lncRNA-Ror regulate autophagy via miR-188-3p and miR-133; *Chrf* stimulates hypertrophy via miR-489; and *MALAT1* reduces cardiac damage by sponging miR-320, miR-204, and miR-150. Regarding circRNAs, *Hrcr* reduces pathological hypertrophy via miR-223, *Cdr1as* exacerbates MI-induced apoptosis via miR-7a, and *circ-Amotl1* protects against CVD by enhancing pAkt nuclear translocation (Ma and Liao, 2019). Despite the therapeutic potential of these ncRNAs in CHF, a significant research gap remains regarding their roles in physiological hypertrophy or EIC (Ma and Liao, 2019).

**Figure 2:**
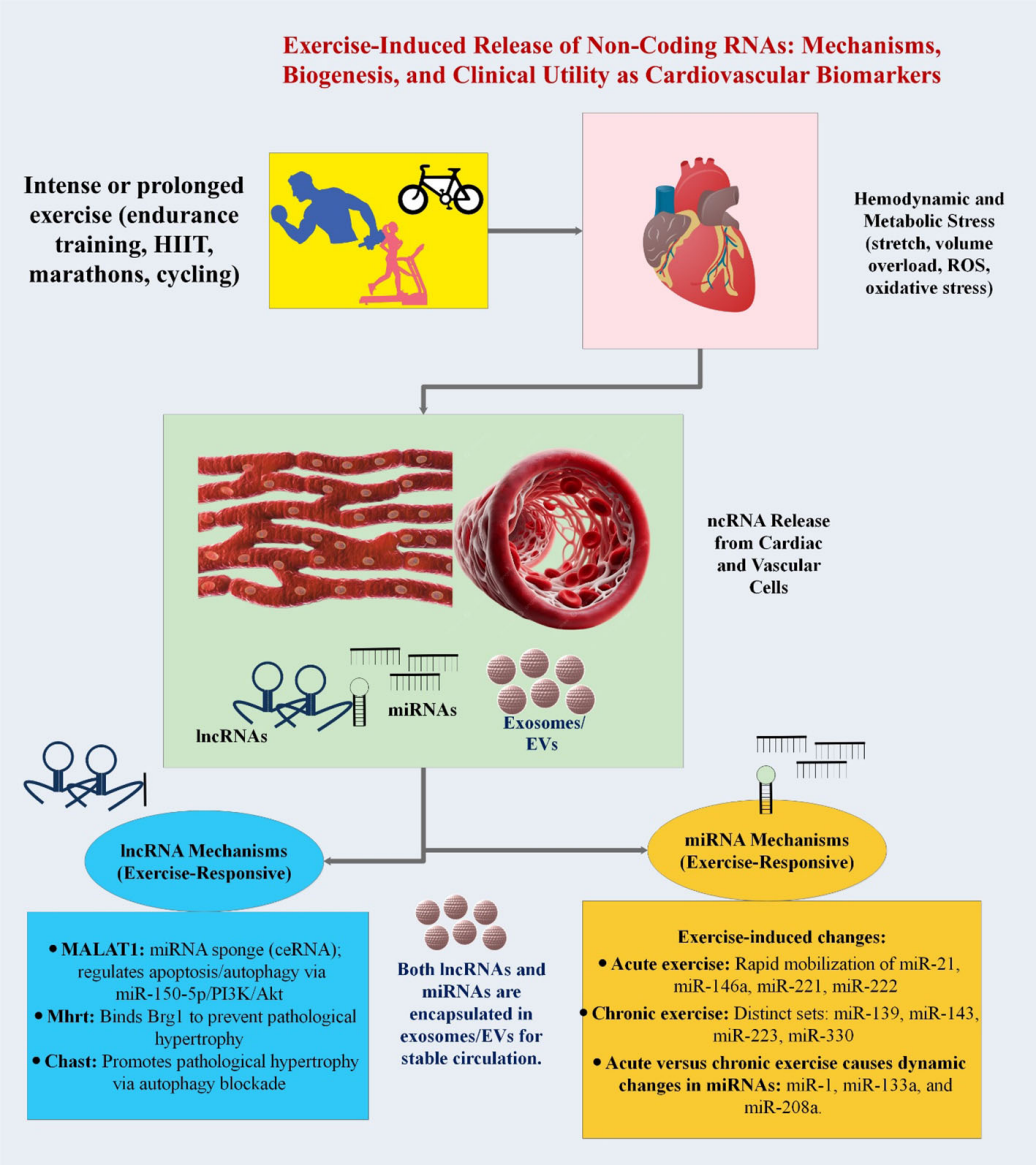
NcRNAs, as cardiovascular biomarkers, are released during exercise. Exercise that is intense or prolonged causes metabolic and hemodynamic stress, thereby stimulating the release of ncRNAs from cardiac and vascular cells. Exercise-responsive lncRNAs (*MALAT1*, *Mhrt*, *Chast*) serve as transcriptional controllers, ceRNAs, and epigenetic regulators (Lee et al., 2003; Zeng and Cullen, 2003; Lee et al., 2004; Faller and Guo, 2008; Morlando et al., 2008). While chronic exercise modifies different sets of miRNAs (miR-139, miR-143, miR-223, miR-330), acute exercise quickly mobilizes miRNAs (miR-21, miR-146a, miR-221, miR-222) (Ultimo et al., 2018). EXOs and EVs encapsulate both miRNAs and lncRNAs to ensure their steady circulation. Early subclinical damage identification, left ventricular hypertrophy type differentiation, and hypertrophic cardiomyopathy screening are examples of clinical value that remain in the exploratory stage and require validation (de Gonzalo-Calvo et al., 2018; Barber et al., 2019; Ma and Liao, 2019; Hu et al., 2021, 2026).

The identification of minimally invasive cardiovascular biomarkers is enabled by miRNAs, proteins, and lipids produced by EXOs and extracellular vesicles (EVs). However, the repeatability of EV datasets is limited by pre-analytic variables and non-standardized procedures. While most evidence remains exploratory (small cohorts, n<50), artificial intelligence (AI) can integrate multi-omics data for feature selection and illness subtyping; ensemble approaches (Random Forest, gradient boosting) perform well (AUC 0.80–0.92). Locked-in assays, multicenter validation (Minimal Information for Studies of EVs (MISEV)-aligned), comparison with validated biomarkers, and transparent AI reporting are necessary for clinical application (Dinc and Ardic, 2026) (**Table 2**; **Figure 2**).

**Table 2:** Overview of principal studies on emerging novel exercise-related CVD biomarkers.

Biomarker category	Study population and intervention	Key finding	Clinical implications	Limitations	Ref.
H-FABP	47 patients (26 with >70% stenosis, 21 normal coronary anatomy), EST	From baseline to three hours after EST, H-FABP dropped (CAD: -2.79 ng/mL; control: -0.84 ng/mL). reduction linked to proteinuria brought on by exercise rather than heart injury.	Proteinuria is a confounding factor; a reduction in H-FABP during EST should not be mistaken for the lack of CAD.	Small sample (n=47); single center; no comparison with troponin; unexpected finding requires replication.	(Arı et al., 2011)
H-FABP, sST2	98 participants, 8 months of regular physical activity	After prolonged exercise, sST2 increased by 793 pg/mL (p=0.045), indicating adaptive vascular stress, whereas H-FABP decreased by 0.57 ng/mL (p<0.01), indicating better perfusion.	Frequent exercise improves indicators of subclinical ischemia; an increase in sST2 may indicate adaptive vascular remodeling.	No control group; single time point post-training; lack of imaging correlation; moderate sample size.	(Sponder et al., 2019)
cMyBP-C	158 participants (75 men, 83 women), exercise stress echocardiography + 1–1.5 year follow-up	Before stress, HR 8.1 for significant occurrences (p=0.041) is the MyBP-C threshold (96% sensitivity). AUC 0.91, HR 13.8 (p=0.000472) for important events (death, MI, stroke, and PE). EF was normal for the majority of crucial occurrences (6/7).	Even in individuals with normal EF, cMyBP-C is a very sensitive screening biomarker for severe CVD.	Small sample (n=158); short follow-up (1–1.5y); no external validation; no CMR comparison.	(Tong et al., 2017)
cMyBP-C	205 symptomatic HFrEF patients (SMARTEX *post hoc* analysis), 12 weeks HIIT or MCT *vs.* control	There was no discernible difference between the groups’ cMyBP-C alterations (Δ IG: -0.5 *vs.* CG: -0.7). There was a correlation between log cMyBP-C and log hs-cTnI (R = 0.52, 95% CI 0.37-0.66, P<0.001).	In HFrEF, cMyBP-C may indicate subclinical myocardial damage during ET.	*Post hoc* analysis; IG merged; no healthy control group; correlation moderate.	(Riveland et al., 2025)
miRNAs	20 CAD patients (10 male, 10 female), maximal cycle ergospirometry	After exercise, 33 miRNAs were altered, with 16 exhibiting sex differences. The responses to nine miRNAs (let-7e-5p, miR-1, miR-19b-1-5p, etc.) varied statistically by sex.	Personalized risk classification in CAD may be made possible by sex-specific miRNA responses to exercise.	Very small sample (n=20); single exercise session; no validation cohort; no long-term outcomes.	(Mayr et al., 2021)
miRNAs	20 sedentary individuals (HERITAGE Family Study), 20 weeks endurance training	Nine of the 14 c-miRNAs were downregulated, and five were upregulated. Target genes that are connected to more than 345 biological processes.	Widespread alterations in c-miRNA patterns associated with metabolic and cardiovascular pathways are produced by regular exercise.	Small sample (n=20); no control group; no correlation with clinical outcomes; descriptive only.	(Barber et al., 2019)
c-miRNAs	Healthy active runners, 10km, half-marathon, and marathon races	Exercise duration led to a dose-dependent increase in all cardiac biomarkers. After ten kilometers, five c-miRNAs were dysregulated, and after a marathon, nineteen. “Pseudo-disease” signatures were brought on by exercise.	Clinicians must identify exercise-induced pseudo-disease patterns, even if c-miRNAs may enhance the diagnostic utility of hs-cTnT/NT-proBNP.	Healthy subjects only; no pathological controls; single time point post-exercise; no follow-up.	(de Gonzalo-Calvo et al., 2018)
miR-15a-5p	ApoE^-/-^ mice (high-fat diet) + moderate treadmill training, 12 weeks	In atherosclerotic aortas, exercise dramatically decreased miR-15a-5p. Through 3’-UTR targeting, miR-15a-5p inhibited *Sema3A*. Plaques were destabilized by VSMC-specific overexpression. Human carotid plaques are positively associated with plaque vulnerability.	A possible therapeutic target for plaque stabilization by AE is miR-15a-5p.	Animal model; little human validation (plaque samples only); no human exercise intervention.	(Hu et al., 2026)
Exosomal miRNAs	Review of exercise-induced EXO studies (HIIT, endurance training)	HIIT targets CTGF and lessens cardiac fibrosis by increasing circulating exosomal miR-133a (2-3×). Exosomal miRNAs control vascular flexibility, cardiac healing, and endothelial function.	Exosomal miRNAs are both promising precision diagnostic tools and mediators of EIC.	Review article; lack of clinical validation; inconsistent methods; absence of standardized tests.	(Yu et al., 2026)
Exosomal EVs + AI	Review of EV biomarker studies (AMI, HF)	AUCs of 0.80–0.92 are achieved for EV biomarker classification using AI ensemble techniques (Random Forest, gradient boosting). The majority of research lacks external validation and has small cohorts (n<50).	Although promising, AI-integrated EV analysis is still in the proof-of-concept stage; multicenter validation linked with MISEV is necessary for clinical adoption.	Exploratory studies only; small sample sizes, no comparisons with recognized biomarkers, and no standardized procedures.	(Dinc and Ardic, 2026)
LncRNA *MALAT1*	Rat model of CHF (left anterior descending ligation), 8 weeks AE post-surgery	Exercise enhanced autophagy and decreased ROS, inflammatory cytokines, MMPs, and apoptosis. Exercise benefits were eliminated by *MALAT1* overexpression. *MALAT1* suppresses PI3K/Akt and downregulates miR-150-5p.	A possible therapeutic target, *MALAT1*, is a crucial modulator of EIC in CHF.	Animal model only; no human validation; only one lncRNA was examined; mechanistic rather than translational.	(Hu et al., 2021)
LncRNAs and CircRNAs	Review of ncRNAs in cardiac hypertrophy and CHF	Autophagy, apoptosis, and hypertrophy are regulated by *Bvht*, *Mhrt*, *Chast*, *Chaer*, *Carmen*, *Fendrr*, *Apf*, *MALAT1*, *Chrf*, *Hrcr*, *Cdr1as*, and *circ-Amotl1*. Important research gap: function in physiological hypertrophy or EIC.	Although ncRNAs offer therapeutic potential for CHF, little is known about how they contribute to exercise-mediated protection.	Review article; mostly preclinical; no evidence on human exercise; descriptive rather than mechanistic for EIC.	(Ma and Liao, 2019)

#### 4.3.4 Summary of evidence gaps and limitations for cardiac-specific ncRNAs

Although there are several limitations, research on exercise-induced ncRNAs (miRNAs, lncRNAs, and circRNAs) shows promise. The majority of research has small sample sizes (n=20–20), uses animal models, or lacks external validation. Significant differences in exercise regimens (modality, duration, intensity) and miRNA panels (5–187 targets) make cross-study comparison difficult. Inconsistent sample collection and standardization practices have a major impact on reproducibility. Despite the identification of “pseudo-disease” signs, there are no recognized cutoffs to distinguish between real pathology and benign exercise-induced alterations. Most research on lncRNAs and circRNAs remains preclinical and descriptive, and little is known about their roles in EIC. With tiny cohorts (n<50) and no comparison to known biomarkers, the EXO-AI area is still at the proof-of-concept stage. Large multicenter cohorts, standardized procedures, sport-specific reference ranges, long-term monitoring, and validation against NPs and hs-troponin for clinical usage are all necessary for future study.

## 5 Diagnostic performance: sensitivity, specificity, and cut-off considerations

### 5.1 Overview of exercise-induced biomarker elevations

The use of biomarkers (cTn, BNP, NT-proBNP, D-dimers, CK-MB, and Mb) to assess cardiovascular function during exercise is expanding, although interpretation remains challenging due to conflicting results across studies. Intense exercise may elevate these markers over pathological thresholds even in healthy persons, making it more difficult to identify acute coronary syndrome, pulmonary embolism, or HF in an emergency (Costache et al., 2022a). CK-MB and Mb are more responsive to physical activity due to skeletal muscle tension (peaking within days and then returning to baseline), whereas cTnT also increases with strenuous activity, though its significance remains debated (Costache et al., 2022a).

### 5.2 Diagnostic accuracy of Exercise Stress Testing

With ischemic ECG abnormalities, EST achieves 86.2% sensitivity and 86.4% specificity, yielding a high diagnostic accuracy (up to 86.3%) for identifying coronary microvascular dysfunction (CMD). Exercise-induced chest pain alone adds limited value (76.1% accuracy) beyond ECG changes. Exercise hemodynamics can identify HF with preserved EF when resting measures are normal, and Cardiopulmonary Exercise Testing (CPET) provides superior evaluation of cardiac and pulmonary function, with a sensitivity of 66–74% for detecting myocardial ischemia compared with gold-standard perfusion imaging. EST correctly distinguishes between structural and functional CMD, with structural abnormalities being observed more frequently. Differentiating between pathogenic changes and exercise-induced cardiovascular remodeling in athletes is a major therapeutic hurdle. Inadequate heart rate response or a low rate-pressure product during exercise is a high-risk indicator. Significant drawbacks include the fact that EST often overlooks functional CMD and that combining many symptoms (such as chest pain and ECG abnormalities) raises specificity to 87.7% but lowers sensitivity to 75.0% (Tsai et al., 2025).

### 5.3 Sensitivity and specificity of established cardiac biomarkers

Exercise-induced cardiovascular biomarkers vary widely in diagnostic efficacy; during severe activity, several cardiac-specific markers show brief, non-pathological increases that should be interpreted with caution. After intensive exercise such as a marathon, cTnI and cTnT, the gold standards for myocardial damage, frequently exceed the 99th percentile but typically return to normal within 24–72 hours without evidence of necrosis. In long-term follow-up, elevations of cTnI >0.040 μg/L have been linked to an increased risk of major adverse cardiovascular events (Celeski et al., 2024). NT-proBNP and BNP rise after vigorous exercise and return to baseline within 72 hours; exercise-induced hypertension (EIH) may be detected using these markers. After intense exercise, CK-MB levels increase but usually reflect skeletal muscle rather than cardiac damage. Mb has limited specificity but peaks early. Emerging biomarkers such as sST2 and Gal-3 reflect fibrosis and mechanical stress. Exercise increases NT-proBNP and SOD while decreasing nitric oxide (NO) response in EIH (defined as systolic blood pressure ≥210 mmHg in males), which presents three diagnostic challenges: 1) less-trained runners show larger elevations than well-trained athletes; 2) sampling timing is crucial (peak values typically occur at 3–4 hours post-exercise) (Stadiotti et al., 2021; Costache et al., 2022b; Celeski et al., 2024; Kim et al., 2025). It is advisable to perform repeated assessments to differentiate between transient and pathological increases, interpret laboratory data within 24 hours after recent activity, and combine CPET with biomarker evaluations to achieve the best cardiovascular risk assessment in athletes (Stadiotti et al., 2021; Costache et al., 2022b; Celeski et al., 2024; Cheng et al., 2025; Frandsen et al., 2025; Kim et al., 2025).

### 5.4 Diagnostic performance of emerging biomarkers

Exercise-related cardiac biomarkers, such as cTn, BNP/NT-proBNP, Gal-3, ST2, H-FABP, cMyBP-C, and ncRNAs, have varying diagnostic performance based on the clinical context (acute *vs.* chronic exercise; healthy athletes *vs.* patients with known CVD) (Shave et al., 2002, 2010; Sedaghat-Hamedani et al., 2015). Regarding emerging biomarkers, ST2 exceeds the 35 ng/dL clinical threshold in 92.7% of athletes after exercise, and cMyBP-C shows high sensitivity (96% at a pre-defined threshold) with HRs of 8.1–13.8 for adverse cardiovascular events (Aengevaeren et al., 2019; Martins da Costa et al., 2021; Yildiz and Balci, 2026). Following cardiac damage, H-FABP increases quickly; however, skeletal muscle release and exercise-induced proteinuria complicate this increase (Arı et al., 2011; Sponder et al., 2019). In 67% of ultramarathon runners, the fibrosis marker Gal-3 surpasses diagnostic criteria (Salvagno et al., 2014; Le Goff et al., 2020).

Currently, there isn’t a single biomarker that can distinguish pathological damage from physiological adaptation. Specificity may be increased by a multi-marker strategy, such as combining cTn, BNP, and Gal-3, although this requires validated algorithms (Ozyildirim et al., 2023; Ianos et al., 2024; Asta et al., 2025).

### 5.5 The problem of standard cutoffs in athletic populations

Sensitivity is often high for detecting myocardial stress or damage following extreme endurance exercise, with high-sensitivity cTn (hs-cTn) showing >90% of athletes exceeding the 99th percentile after a marathon (Javed et al., 2010; Scherr et al., 2011). Standard AMI cutoffs, however, have poor specificity when applied to athletic populations: 76–96% of healthy athletes fulfill MI criteria for hs-cTn within 3 hours post-race, resulting in unacceptably high false-positive rates (Wedin et al., 2020; Mair, 2025). Since the 99th-percentile URL derived from sedentary populations is unsuitable for athletes, sport-specific reference ranges are urgently needed (Santos et al., 2014; He et al., 2022). To evaluate rise-and-fall patterns, current recommendations advocate serial sampling (baseline, 3 hours, and 24 hours post-exercise); a continuous elevation beyond 24 hours or the absence of a decrease implies an underlying illness rather than a benign exercise response (Thygesen et al., 2018; Pelliccia et al., 2021).

### 5.6 Confounding factors and considerations for cutoff determination

Age, fitness level, kind and duration of exercise, sex differences (women show larger post-exercise biomarker elevations than males), and renal function are significant confounding variables that need to be taken into account when setting cutoffs (Baker et al., 2016; Andrade et al., 2021; Ferrera et al., 2025). Future studies need to concentrate on: 1) establishing 99th percentile URLs particular to each sport and sex; 2) verifying point-of-care (POC) devices in field situations; and 3) building integrated diagnostic algorithms that take into account serial biomarker measures, imaging data, and clinical presentation (Regan et al., 2021; Nazir et al., 2025).

## 6 Clinical applications

### 6.1 Overview of clinical scenarios for exercise-related cardiac biomarkers

The clinical application of exercise-related cardiac biomarkers focuses on three main scenarios: (1) assessing acute cardiac volume or pressure overload; (2) distinguishing between physiological athlete’s heart and pathological cardiomyopathies; and (3) early detection of hidden cardiovascular conditions. Temporary elevations in high-sensitivity cTn (hs-cTn), BNP/NT-proBNP, and H-FABP are often observed in the setting of acute overload, such as during endurance events like marathons or ultratriathlons, indicating reversible cardiac stress rather than actual tissue death. Serial blood tests taken at baseline, three hours, and 24 hours after exercise can differentiate benign biomarker release from acute coronary syndrome; a return to normal levels within 24 to 72 hours usually reflects physiological adaptation rather than myocardial damage (Scherr et al., 2011, 2015).

### 6.2 Exercise after stroke: cardiac and functional outcomes

In a single-masked RCT, high-intensity AE and low-intensity balance/flexibility (BF) training were compared over 6 months (3 sessions per week, 60 minutes each) in 50 individuals (age 50–80 years, >1 year post-stroke). The primary outcome, V̇O_2peak_, did not significantly change in either group (AE: 16.9 to 17.4 *vs.* BF: 16.9 to 16.6 mL·kg^-1^·min^-^^1^, p=0.45). Right atrial emptying fraction, however, significantly improved in the AE group (AE: 30% to 37% *vs.* BF: 35% to 31%, p=0.04), suggesting improved right-sided cardiac function and early myocardial relaxation. Lipid profiles, glucose, homocysteine levels, and ambulatory ability all showed substantial improvements in both groups (p<0.04). The BF group had one non-injurious fall; no other adverse events were noted. The investigators determined that whereas LES equally promotes metabolic indicators and mobility, HES improves right atrial function. This discovery provides essential insights into the exercise-induced mitigation of cardiovascular risk after a stroke (Tang et al., 2014).

### 6.3 Dual-biomarker approaches for detecting myocardial ischemia

To determine whether combining hs-cTnI and BNP improves the noninvasive detection of exercise-induced myocardial ischemia compared with single biomarkers alone, 1,142 consecutive patients with suspected myocardial ischemia referred for stress myocardial perfusion imaging (MPI) were included in the study. Of these, 456 individuals (40%) had inducible myocardial ischemia. The AUC for identifying ischemia was 0.66 for clinical judgment before stress testing (CJb), 0.70 (p=0.07 *vs.* CJb) for hs-cTnI alone, and 0.66 (p=0.98) for BNP alone. Compared to hs-cTnI plus CJb, a dual-biomarker approach combining hs-cTnI and BNP with CJb did not substantially increase diagnostic accuracy (AUC 0.74 *vs.* 0.74, p=0.16). Regarding prognosis, BNP predicted mortality (HR 1.6) during follow-up, while hs-cTnI predicted AMI (HR 1.6). The researchers determined that, in addition to clinical judgment and hs-cTnI alone, a dual-biomarker strategy including BNP and hs-cTnI did not provide supplementary diagnostic value (NCT01838148) (Walter et al., 2020).

### 6.4 Release kinetics of troponin subtypes after endurance exercise

Researchers investigated the release kinetics of hs-cTnI and hs-cTnT after endurance exercise in 25 healthy participants who completed a 30-kilometer run. The researchers examined whether remote ischemia preconditioning (RIPC), consisting of four 5-minute intervals of unilateral occlusion at 220 mmHg, reduced exercise-induced cTn release. The run significantly elevated all cTn levels (p<0.001), with mean peak concentrations of 47 ± 27 ng/L for hs-cTnT, 69 ± 62 ng/L for hs-cTnI, and 82 ± 64 ng/L for s-cTnI. Significantly, 60% of individuals exhibited peak hs-cTnT levels two hours post-exercise, whereas 84% and 80% demonstrated peak hs-cTnI and s-cTnI levels five hours thereafter. RIPC did not diminish exercise-induced cTn release (time × trial: all p>0.5). The researchers determined that exercise-induced hs-cTnT levels precede those of hs-cTnI, whereas in AMI, both troponins increase simultaneously. The unique release kinetics and the absence of RIPC effects support the notion that exercise-induced cTn release is mechanistically distinct from cTn release in AMI (Klinkenberg et al., 2016).

### 6.5 Effects of endurance training on baseline and post-exercise biomarkers

Investigators assessed the impact of a 14-week endurance running program on exercise-induced release of NT-proBNP and hs-cTnT in 58 untrained participants who were randomly assigned to either a control group or a supervised endurance exercise group (3–4 days/week, 120–240 min/week, 65–85% of maximal heart rate). Acute exercise considerably increased hs-cTnT in both groups before training (p<0.0001), with 71% of individuals above the URL. Both baseline and post-exercise hs-cTnT levels were considerably higher after training compared to both the pre-training and control groups (p=0.008). NT-proBNP increased slightly but significantly after acute exercise, although training did not affect this response (p=0.121) (Legaz-Arrese et al., 2015). The scientists discovered that a 14-week endurance training regimen elevated hs-cTnT levels both pre- and post-exercise, but no training-related alterations in NT-proBNP were seen (Legaz-Arrese et al., 2015).

### 6.6 Influence of exercise modality and menstrual cycle on cTnT release

In a randomized crossover study involving 17 healthy eumenorrheic women, researchers compared exercise modality, moderate-intensity continuous exercise (MCE) (60% V̇O_2max_ steady-state cycling until 300 kJ) versus work-equivalent high-intensity interval exercise (HIE, repeated 4-minute cycling at 90% V̇O_2max_ interspersed with 3-minute rest), and menstrual cycle phase (follicular phase *vs.* luteal phase) on exercise-induced cTnT elevation. In all modalities, cTnT dramatically rose after exercise (MCE at 3 and 4 hours; HIE at 1, 3, and 4 hours). With median peak levels ranging from 4.8 to 8.2 ng/L, there were no statistically significant variations in peak post-exercise cTnT (mainly at 3 hours) among the four trials (Nie et al., 2020). A single 300 kJ session of either MCE or HIE considerably increases cTnT levels, with cTnT elevation occurring somewhat sooner after HIE; however, no differences were observed across menstrual cycle phases or exercise modalities (Nie et al., 2020).

### 6.7 Very early exercise after ischemic stroke: inflammatory and clinical outcomes

In a prospective, single-masked RCT, 48 patients with mild-to-moderate first-ever ischemic stroke participated. The study examined the effects of VEE, initiated within 24 hours of onset and lasting 45 minutes twice daily for 7 days (1.5 hours/day), on inflammatory markers and clinical outcomes, compared with standard care (regular turning and positioning). A non-linear effect on fibrinogen was observed. Between days 4 and 7, the VEE group tended toward lower levels of IL-6, leukocytes, neutrophils, and monocytes, and higher levels of lymphocytes. The VEE group showed noticeably greater improvements in motor impairment, physical disability, functional independence, anxiety, depression, and cognition at 1- and 3-month follow-ups. Clinical outcomes over time were linked to positive inflammatory modulation by VEE, including significant correlations between changes in IL-6 at days 4 and 7 and 3-month functional independence (rs = -0.33, p = 0.019; rs = -0.33, p = 0.021) and between day 7 IL-6 changes and 3-month motor impairment (rs = 0.30, p = 0.039) (Ademoyegun et al., 2025). Researchers showed that moderate-intensity VEE initiated within 24 hours improves post-stroke physical, motor, cognitive, and emotional recovery and modulates inflammatory markers, particularly IL-6 (Ademoyegun et al., 2025).

### 6.8 Resistance training, blood flow restriction, and circulating miRNAs

As a model of peripheral artery disease, this three-arm, randomized-balanced crossover study examined the effects of acute resistance training on c-miRNAs under three conditions: high-intensity (70% one-repetition maximum (1RM), HI), low-intensity without blood flow restriction (30% 1RM, LI), and low-intensity with blood flow restriction (LI-BFR) (30% 1RM + 300 mmHg cuff, LI-BFR). Heart rate and lactate increased across all conditions (p<0.001), with HES producing the greatest increase in lactate. High-intensity training up-regulated six miRNAs (miR-139-5p, miR-143-3p, miR-195-5p, miR-197-3p, miR-30a-5p, and miR-10b-5p), while LI-BFR down-regulated miR-143-3p. The expression of miR-143-3p was significantly and positively correlated with lactate concentration (r=0.52, p=0.009), and partial correlation analysis suggested that training type (LI-BFR *vs.* high-intensity) had a systematic effect on this association (Vogel et al., 2019). The scientists demonstrated that HES and LI-BFR had a substantial impact on lactate and the arteriogenesis-associated miRNA-143-3p. To increase collateral flow and serve as an external stimulus for arteriogenesis, blood flow restriction may mimic arterial closure (Vogel et al., 2019).

### 6.9 Exercise-induced EVs and intercellular communication

Researchers used quantitative proteomics to characterize the exercise-induced synthesis of proteins present in EVs from healthy individuals after a 1-hour cycling session. EXO and tiny vesicle components were significantly enriched in the circulation of over 300 proteins after exercise. Pulse-chase and intravital imaging experiments showed that exercise-released EVs transported their protein cargo and preferentially localized to the liver. Additionally, arteriovenous balance tests throughout the contracting human leg revealed several other possible myokines released into circulation independently of traditional secretion pathways. These findings, according to the researchers, show a unique method by which tissue crosstalk during exercise uses EV-mediated intercellular communication to generate systemic biological effects (Whitham et al., 2018).

### 6.10 Summary of evidence gaps and limitations in clinical applications

Several important issues hamper research on the therapeutic use of exercise-related cardiac biomarkers. Although the larger MPI trial (n=1,142) was well-powered, it demonstrated that multi-marker techniques do not always improve prediction performance, as adding BNP to hs-cTnI did not confer any significant diagnostic advantage over hs-cTnI alone. The small sample size in the majority of studies, between 17 and 58 for exercise trials and 48 for the stroke investigation, is a significant drawback. Significant heterogeneity among study populations, which include women at various stages of the menstrual cycle, stroke patients, healthy individuals, and those with suspected ischemia, further restricts generalizability, especially since no direct comparisons between these groups have been carried out. The short follow-up time, which ranges from a few hours to six months, is another disadvantage. This leaves open the question of long-term outcomes, particularly whether exercise-induced biomarker elevations predict significant adverse cardiac events years later. The stroke experiment showed positive inflammatory modulation but lacked a genuine no-exercise control group and yielded relatively modest correlations (rs = -0.33 to 0.30), suggesting that inflammation may account for only a portion of the variance in recovery. Additionally, the blood flow restriction miRNA study was underpowered, and the proteomics/EV research remained descriptive without confirmation in larger cohorts or correlation with clinical outcomes.

The lack of multicenter-validated, sport-specific reference ranges, prospective validation of biomarker-informed return-to-play decisions, and direct comparisons between advanced cardiac imaging and biomarkers in athletes with suspected cardiomyopathies are among the major evidence gaps that remain. Large, multicenter prospective studies with long-term follow-up (5–10 years), the adoption of standardized pre-analytical procedures, and the validation of integrated algorithms that integrate biomarkers, imaging, and clinical assessment to enable customized risk stratification must be given top priority in future research to address these problems.

Exercise-related biomarker results in particular groups provide supplementary insights outside athletic populations. ET enhances RV systolic function in Chronic Obstructive Pulmonary Disease (COPD) patients, and NT-proBNP is correlated with right heart strain and pulmonary hypertension severity (Şahin Özdemirel et al., 2014; Aritakulu Badrinath et al., 2025; Avdeev et al., 2025). HES improves right atrial function in stroke patients, and both high- and low-intensity training improve metabolic indicators (Tang et al., 2014). VEE (within 24 hours) promotes recovery and positively controls inflammatory markers, especially IL-6 (Ademoyegun et al., 2025). Gal-3, NT-proBNP, and hs-TnI are temporarily elevated during SCUBA diving; different recovery kinetics indicate transitory circulatory adaptation (Žarak et al., 2020). Similar to high-intensity training, blood flow restriction training, in conjunction with low-intensity resistance exercise, affects the arteriogenesis-associated miRNA-143-3p (Vogel et al., 2019). LES lowers cortisol and oxidative stress in sleep-deprived people, but HES increases these detrimental effects (Park et al., 2023). These results demonstrate the context-dependent nature of exercise-induced biomarker responses and the significance of customized exercise prescription, even when limited to specific populations. **Table 3** provides further information.

**Table 3:** Biomarker responses to exercise in special populations. Studies on COPD, stroke, diving, blood flow restriction training, and sleep deprivation, briefly mentioned in the main text, are summarized here for completeness.

Population	Study design	Sample size	Intervention	Key biomarker findings	Clinical implication	Ref
COPD	Observational	Not specified (review article)	ET in COPD patients	NT-proBNP correlates with right heart strain and pulmonary hypertension severity; ET improves RV systolic function by echocardiography	A certain threshold may identify early cardiovascular damage in COPD; NT-proBNP predicts a poorer prognosis and future exacerbation risk.	(Şahin Özdemirel et al., 2014; Aritakulu Badrinath et al., 2025; Avdeev et al., 2025)
Post-Stroke	Single-masked RCT	50 patients (age 50-80, >1 year post-stroke)	6 months high-intensity AE *vs.* low-intensity BF training (3×/week, 60 min)	Right atrial emptying fraction improved in the AE group (30%→37% *vs.* BF: 35%→31%, p=0.04); lipids, glucose, and homocysteine improved in both groups (p<0.04)	Both forms of exercise enhance metabolic indicators, and HES improves right atrial function after a stroke.	(Tang et al., 2014)
Post-Stroke (Very Early)	Prospective single-masked RCT	48 patients with mild-to-moderate first-ever ischemic stroke	VEE initiated within 24h (45 min twice daily ×7 days) *vs.* standard care	VEE reduced IL-6, leukocytes, neutrophils, monocytes; increased lymphocytes (days 4-7); 3-month outcomes correlated with IL-6 changes (rs = -0.33 to -0.33, p=0.019-0.021)	Within a day, moderate-intensity VEE enhances recovery and effectively controls inflammatory markers, particularly IL-6.	(Ademoyegun et al., 2025)
SCUBA Diving	Observational	16 male recreational divers	30-minute SCUBA dive to 30 meters	Gal-3, NT-proBNP, hs-TnI, Mb, and VEGF increase immediately post-dive; Gal-3 and Mb returned to baseline; NT-proBNP and hs-TnI continued rising during recovery	Changes in biomarkers indicate a transient adaptation of the muscular and circulatory systems to diving; the long-term effects are unclear.	(Žarak et al., 2020)
Blood Flow Restriction (BFR) Training	Three-arm randomized crossover	Not specified (crossover design)	High-intensity (70% 1RM) *vs.* low-intensity without BFR (30% 1RM) *vs.* LI-BFR (30% 1RM + 300 mmHg cuff)	High-intensity up-regulated 6 miRNAs (miR-139-5p, -143-3p, -195-5p, -197-3p, -30a-5p, -10b-5p); LI-BFR down-regulated miR-143-3p; miR-143-3p correlated with lactate (r=0.52, p=0.009)	Arteriogenesis-associated miRNA-143-3p is impacted by both LI-BFR and HES; BFR may mimic arterial obstruction to increase collateral flow.	(Vogel et al., 2019)
Sleep Deprivation	Experimental	Not specified (crossover design)	Low-intensity (LES) *vs.* high-intensity (HES) AE after 3 days of sleep deprivation	SD increased cortisol, hs-CRP, and oxidative stress (D-ROM); LES reduced cortisol and oxidative stress; HES further increased both; no significant change in hs-CRP after any exercise intervention	For those who are sleep deprived, LES is suitable and safe; HES intensifies adverse consequences.	(Park et al., 2023)

## 7 Future directions

Despite tremendous advancements, several challenges remain in the clinical use of exercise-related cardiac biomarkers. Small sample sizes, brief follow-up periods, and a variety of research methodologies characterize the present database, which remains largely exploratory. As a result, future research priorities are outlined in the parts that follow, along with reasonable timeframes and an explicit recognition of current constraints.

### 7.1 Current limitations in biomarker interpretation for exercise monitoring

The use of biomarkers to track exercise-induced cardiovascular stress has many major challenges. The substantial inter-individual variability in biomarker responses to exercise, largely due to genetic variation (e.g., in creatine kinase (CK) owing to polymorphisms affecting sarcolemmal stability), is a major challenge. As a result, population-averaged responses often fail to capture individual regulatory profiles. This absence of personalized reference data makes it difficult to interpret individual measurements (Hacker et al., 2023; Kasiak, 2026).

Furthermore, while biomarkers such as IL-1RA, IL-6, cortisol, CK, and lactate dehydrogenase indicate tissue disturbance, metabolic stress, or immune responses, their predictive value for regeneration or recovery, specifically, their ability to anticipate how an athlete will respond to future training loads, remains largely unclear. Instead of demonstrating individual predictability, which is an essential precondition for practical use in load management, most research focuses on group-level kinetics (Hacker et al., 2023; Kasiak, 2026). Additionally, the return-to-baseline regulation of these molecules after exercise cessation is poorly understood at the individual level, limiting their use in monitoring differential regeneration and adaptation. Therefore, even when candidate markers are available, the conversion of biomarker measurements into useful, tailored training recommendations is hampered by insufficient knowledge of individual regulatory patterns, genetic heterogeneity, and the lack of validated predictive models for recovery and performance outcomes (Hacker et al., 2023; Kasiak, 2026).

### 7.2 Post-translational modifications and personalized exercise prescription

The study of exercise-specific PTM expression profiles has greatly advanced our understanding of the molecular mechanisms underlying physical activity. These profiles offer a mechanistic framework for differentiating responses to aerobic, resistance, and HIIT. Important concepts include the explanation of PTM-mediated metabolic memory and the “PTM threshold” theory, which connect exercise intensity to metabolic adaptation and provide a quantifiable method for understanding how different exercise loads confer long-term health benefits. The application of ML to PTM fingerprint profiles facilitates the creation of individualized training regimens and simplifies the analysis of complex data (Shen et al., 2026).

These theoretical developments advance the emerging field of exercise pharmacology, which may offer therapeutic advantages for uncommon illnesses and address inter-individual variability in exercise responses. However, there are still issues, including methodological variability, ML biases (e.g., training-dataset constraints), and the complexity of PTM interactions with other molecular networks (Shen et al., 2026). To identify PTM-based risk signatures, future research should focus on integrating multi-omics datasets, conducting thorough longitudinal clinical trials, and developing population-scale baseline PTM atlases spanning the health-to-disease continuum. Subject to prospective validation and standardized pre-analytics, this framework ultimately supports a prevention-first model that transforms exercise prescription for disease management and health maintenance by utilizing baseline PTM profiles to inform tailored exercise prescriptions and monitoring strategies (Shen et al., 2026).

### 7.3 Machine learning and multi-omics integration

The complex, high-dimensional data produced by exercise biomarker studies, such as cTn, NPs, Gal-3, ST2, H-FABP, cMyBP-C, and ncRNAs, are increasingly being analyzed using ML and multi-omics techniques (Su and Chen, 2025; Zheng et al., 2025; Greco et al., 2026; Liu et al., 2026; Shen et al., 2026).

The evidence that is currently available is still preliminary. For EV-derived biomarker panels in AMI and HF, ensemble ML approaches such as Random Forest and gradient boosting achieve decent classification performance, with AUCs of 0.80 to 0.92. Nevertheless, most research lacks external validation and uses small cohorts of fewer than 50 individuals (Dinc and Ardic, 2026).

Clinical preparedness has not yet been determined. In the future, ML models that integrate clinical metadata, serial biomarker measurements, imaging data from CMR and echocardiography, and CPET parameters may predict the risk of adverse events, distinguish pathological remodeling from physiological adaptation, and inform athletes’ return-to-play decisions. These applications are still being researched.

Many significant obstacles hamper clinical translation. These include the need for explainable AI (XAI) to guarantee clinical interpretability, the lack of sport-specific reference ranges, the lack of prospective validation in large multi-center cohorts, the need for federated learning to facilitate multi-site data integration, and the requirement for regulatory-aligned locked-in assay designs (Deligiannis et al., 2025; Greco et al., 2026; Liu et al., 2026).

Instead of months, a realistic schedule for clinical deployment will probably take years, or perhaps decades. Standardized practices, sufficiently powered validation studies, and regulatory alignment are necessary to achieve broad clinical usage (Dinc and Ardic, 2026).

### 7.4 A three-stage framework for biomarker development

There are three steps involved in developing exercise-linked biomarkers. First, potential markers are found using omics approaches in the discovery stage. Second, repeated measurements are used in the verification step to verify the identification, expression levels, sensitivity, specificity, and repeatability of each marker. Third, before widespread use, clinical validation, also known as qualification, evaluates the trustworthiness of indicators in similar therapies to determine their clinical value (Haller et al., 2023).

Standardized test-retest designs are crucial for assessing biomarker responses to acute loads and for evaluating repeatability. These designs require sufficient post-exercise observation times to determine biological half-life and re-regulation kinetics. Dose-response insights are enabled by accurate participant characterization that accounts for environmental confounders, high *vs.* low responder status, and external load measurements. Because longitudinal study designs can capture both acute and long-term responses to treatments, such as training camps, they are especially useful for evaluating a biomarker’s ability to “forecast” outcomes such as disease or injury. To determine the suitability of a biomarker panel for exercise load management, repeated blood sampling at rest and after exercise, combined with established monitoring tools, helps establish individualized reference values, evaluate sex-specific effects, training-volume influences, and training status (Haller et al., 2023).

### 7.5 Future research directions and practical recommendations

Future studies on exercise-associated cardiac biomarkers must focus on a few key areas to make significant progress:

First, rather than relying on cutoffs derived from sedentary populations (which often produce false positives), we need to develop standardized testing protocols and reference ranges that accurately reflect athletic populations, accounting for factors such as age, sex, fitness level, and sport type.

Second, there is an urgent need for large, multicenter prospective studies with long-term follow-up (5–10 years) to determine whether transient post-exercise biomarker elevations predict future adverse cardiac events.

Third, integrating ML and XAI with multi-omics data (genomics, proteomics, metabolomics) could enable the development of intelligent models that integrate clinical data, serial biomarker measurements, cardiac imaging, and exercise test results to distinguish subclinical heart disease from healthy athletic adaptation.

Fourth, we must adhere to a rigorous three-step biomarker development framework (discovery, verification, clinical validation), using standardized test-retest designs, longitudinal monitoring to establish individual baseline values, and assessment of post-exercise return-to-baseline kinetics.

Fifth, collaborative data sharing via federated learning (which preserves privacy), clinically interpretable AI systems, and regulatory-aligned locked-in assay designs are necessary for real-world implementation.

**Figure 3:**
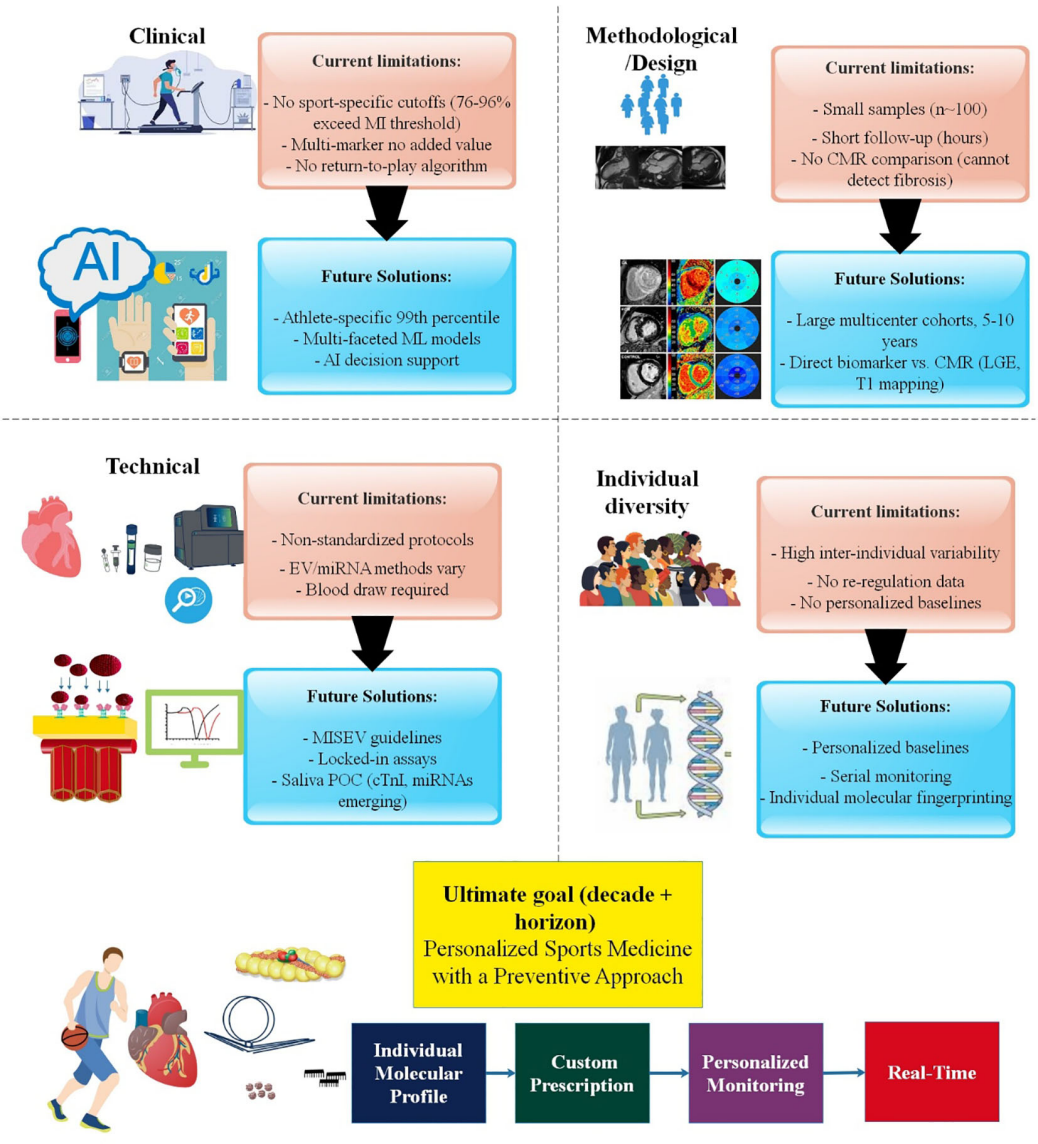
Exercise-induced cardiac biomarkers in four domains: present constraints and potential remedies. Clinical (no sport-specific cutoffs, 76–96% of athletes exceeding myocardial infarction thresholds, multi-marker strategies adding no value, absence of validated return-to-play algorithms); methodological/design (small sample sizes, short follow-up durations, lack of comparison with CMR gold standard); technical (non-standardized pre-analytical protocols, variability in EV and miRNA methods, requirement for invasive blood sampling); and individual diversity (high inter-individual variability, lack of re-regulation data, no personalized baseline reference values). Athlete-specific 99th percentiles, multifaceted ML models, AI-driven decision support, large multicenter cohorts with 5–10 year follow-up, direct biomarker comparison with CMR (T1 mapping and late gadolinium enhancement), MISEV-aligned guidelines, locked-in assays, and saliva-based POC testing (validated for cTnI, emerging for miRNAs) are some of the suggested solutions. Prevention-first personalized sports medicine, which integrates individual molecular profiles, individualized exercise prescriptions, personalized monitoring, and real-time feedback, is the ultimate long-term aim (with a ten-plus-year horizon). Without thorough prospective validation, all potential applications remain hypothetical (Hacker et al., 2023; Su and Chen, 2025; Zheng et al., 2025; Dinc and Ardic, 2026; Greco et al., 2026; Kasiak, 2026; Liu et al., 2026; Shen et al., 2026).

The ultimate goal is a prevention-first approach that transforms exercise medicine from a one-size-fits-all approach to truly personalized treatment for patients and athletes by utilizing each person’s molecular profile to inform tailored exercise regimens and real-time monitoring (**Figure 3**).

### 7.6 Tempered conclusion on future potential

Conceptually, real-time, non-invasive monitoring of exercise-induced cardiovascular stress is enabled by the convergence of biomarker discovery, ML, and tailored digital health platforms. However, without thorough confirmation, promises about real-time monitoring, AI-powered multi-omics, and customized molecular exercise prescription remain theoretical (Deligiannis et al., 2025; Greco et al., 2026; Liu et al., 2026).

The routine use of emerging biomarkers such as ncRNAs and exosomal markers, AI-driven personalized exercise prescription, real-time biomarker monitoring in athletic settings, and biomarker-guided return-to-play algorithms is among the applications that are currently unprepared for clinical practice.

Large multicenter prospective cohorts with five to ten years of follow-up, standardized pre-analytical protocols, sport-specific 99th-percentile reference ranges, prospective validation against hard clinical outcomes, and regulatory approval for any diagnostic claim are all necessary before clinical adoption.

The ultimate long-term goal is a prevention-first approach that uses each person’s unique molecular profile to guide personalized exercise plans and monitoring. However, it will take decades, not just years, of rigorous validation, interdisciplinary collaboration, and regulatory oversight to achieve this objective.

## 8 Conclusion

Exercise-related cardiac biomarkers (cTn, BNP, Gal-3, ST2, H-FABP, cMyBP-C, and ncRNAs) provide important information on myocardial stress and adaptation in athletes. Small sample sizes, short follow-up periods, inconsistent methods, and—most importantly—the improper application of sedentary population cutoffs to athletes (76–96% of healthy marathon runners meet traditional MI criteria post-race) remain significant limitations.

The long-term prognostic significance of repeated biomarker elevations, the ambiguous tissue origins (cardiac *vs.* skeletal muscle) of ST2 and Gal-3 during exercise, contradictory findings (e.g., H-FABP decreasing due to proteinuria versus increasing after injury), and the total lack of sport-specific reference ranges are among the critical evidence gaps that still need to be filled.

Large multicenter prospective cohorts with 5–10 year follow-up, validation of non-invasive POC tests (sweat, saliva), integration of multi-omics with XAI and ML, federated learning for data sharing, and rigorous three-phase biomarker development with standardized protocols are all future priorities. Which thresholds warrant advanced imaging, whether biomarker screening enhances clinical outcomes, and how to integrate biomarkers into well-established diagnostic algorithms are among the major unresolved concerns.

Exercise-induced biomarker increases in asymptomatic athletes should be seen as mostly benign and temporary until these problems are addressed. However, increases that persist for more than 72 hours or are accompanied by symptoms (such as syncope, dyspnea, or chest discomfort) warrant further testing, including echocardiography, serial biomarker monitoring, and advanced cardiac imaging. Although there is potential in the confluence of EXO biology, AI, and multi-omics, clinical implementation will take decades, not years, of thorough validation and interdisciplinary collaboration.
